# Digital Physiotherapeutic Elbow-Specific Training System for Patients After Arthroscopic Release of Elbow Contracture: Noninferiority Randomized Controlled Trial

**DOI:** 10.2196/87459

**Published:** 2026-06-09

**Authors:** Yanmao Wang, Lihua Huang, Ruixin Wang, Zhihao Xu, Yanhong Ma, Shiyang Yu, Jian Ding, Yun Shen, Shengdi Lu

**Affiliations:** 1 Department of Orthopedics Shanghai Sixth People’s Hospital Affiliated to Shanghai Jiao Tong University School of Medicine Shanghai China; 2 Department of Rehabilitation Shanghai Sixth People’s Hospital Affiliated to Shanghai Jiao Tong University School of Medicine Shanghai, Shanghai China; 3 School of Public Health Fudan University Shanghai, Shanghai China; 4 Dong Fureng Institute of Economic and Social Development Wuhan University Wuhan, Hubei China; 5 Pennington Biomedical Research Center Baton Rouge, LA United States

**Keywords:** digital training system, noninferiority, elbow stiffness, arthroscopic release, cost-effectiveness

## Abstract

**Background:**

The effectiveness of a digital training (DT) system in which patients receive individually tailored physiotherapeutic elbow-specific training (PEST) delivered via a digital platform remains unclear.

**Objective:**

This study determines the effectiveness of a DT system in which patients receive individually tailored PEST supervision and guidance via the Joymotion Intelligent Rehabilitation System and educational videos, compared with conventional training (CT) conducted by qualified physiotherapists at outpatient clinics and unsupervised home-based PEST in patients following arthroscopic release for posttraumatic elbow stiffness.

**Methods:**

This single-center, noninferiority randomized controlled trial was conducted at the Rehabilitation Department of Shanghai Sixth People’s Hospital between September 2020 and June 2024. Patients aged 16-65 years undergoing arthroscopic release for posttraumatic elbow stiffness were randomized to receive either a 12-week DT program or conventional outpatient clinic-based training. Outcome measures included elbow flexion-extension range of motion (primary outcome); forearm rotation; isometric and dynamic muscle strength; American Shoulder and Elbow Surgeons (ASES) and Disabilities of the Arm, Shoulder, and Hand (DASH) scores; EQ-5D-5L; cost-effectiveness; adherence; and adverse events, assessed at 4, 12, and 24 weeks postoperatively.

**Results:**

At 12 weeks, the mean elbow flexion-extension range of motion improved similarly in the DT and CT groups (between-group difference –1.6°, 95% CI –8.2° to 4.9°; *P*=.53), confirming noninferiority. Forearm rotation gains were slightly greater with DT (difference 14.2°, 95% CI 2.9°-25.6°). Patient-reported outcomes were equivalent between groups: ASES function (difference 0.6, 95% CI –0.3 to 1.5; *P*=.39) and pain (difference 0, 95% CI –8.3 to 8.5; *P*=.68) subscores, DASH (difference 0.23, 95% CI –1.54 to 1.99; *P*=.68), and EQ-5D-5L index (difference 0.001, 95% CI –0.012 to 0.015; *P*=.56) showed no significant between-group differences. Nearly all patients completed the 12-week program in both arms (104/106, 98.1%, vs 101/102, 99%, adherence; odds ratio 0.52, 95% CI 0.05-5.77). Adverse events occurred in 29 out of 106 (27.4%) participants in the DT group and 32 out of 102 (31.4%) participants in the CT group (odds ratio 0.82, 95% CI 0.45-1.50). Total rehabilitation costs per patient were lower in the DT group by an average of CNY –7418.58, and incremental cost-effectiveness analysis indicated that DT provided comparable outcomes at lower cost.

**Conclusions:**

Individually tailored PEST delivered via a DT system is a viable, cost-effective, and safe alternative to conventional outpatient clinic-based training following arthroscopic release for posttraumatic elbow stiffness. These findings support its integration into routine postsurgical care, particularly for patients facing barriers to traditional therapy.

**Trial Registration:**

Chinese Clinical Trial Registry Chictr2400093415; https://www.chictr.org.cn/showprojEN.html?proj=240693

## Introduction

Digital training (DT) systems, which involve the remote delivery of training services via digital technologies, have emerged as an attractive alternative or supplement to conventional in-hospital therapy. By using mobile apps and videoconferencing, DT enables patients to perform guided exercises at home, reducing the need for frequent hospital visits and overcoming geographic, logistical, and financial barriers. Studies have demonstrated that DT systems can yield outcomes comparable to those of traditional face-to-face interventions in postsurgical recovery settings [1-7].

In the United States and Europe, technological advances combined with supportive health care policies have accelerated the adoption of mobile-based DT. For instance, expanded telehealth coverage by Medicare in the United States and the integration of telemedicine within several European health care systems have facilitated timely, high-quality care delivered remotely [4,5,7]. These measures not only ease the burden of travel for patients but also promote adherence to training protocols through flexible scheduling and continuous monitoring. By contrast, DT systems in China are still in the developmental phase. Although major urban centers are beginning to deploy mobile health apps and telemedicine platforms, the supporting clinical evidence is less robust, and implementation practices vary widely.

Posttraumatic elbow stiffness, a condition marked by reduced range of motion (ROM) due to scar tissue formation following injury, can significantly impair daily activities [8]. Arthroscopic release is commonly used to surgically address elbow contracture by removing fibrotic tissue and improving joint mobility [9]. However, the success of this procedure relies heavily on effective postoperative rehabilitation. This typically involves intensive physiotherapeutic elbow-specific training (PEST) to restore elbow motion and function. Traditional in-hospital rehabilitation offers direct supervision and hands-on therapy; however, it presents several challenges. Frequent hospital visits can be particularly burdensome for patients in rural areas or those with limited mobility. Additionally, rigid scheduling and transportation difficulties may compromise patient adherence, thereby affecting the overall recovery process. DT systems address these challenges by bringing rehabilitation directly to the patient’s home. DT programs provide daily exercise instructions, track adherence, and facilitate real-time, 2-way communication between patients and physical therapists. This model minimizes logistical hurdles and supports continuous, patient-centered care, which is crucial during the early postoperative period, when aggressive rehabilitation is needed to prevent recontracture. Furthermore, DT systems offer the flexibility to adjust exercise regimens in response to patient performance and feedback, closely mimicking the personalized care provided in traditional settings.

While DT systems have been extensively studied in lower limb surgeries, research on their use in upper extremity conditions, specifically posttraumatic elbow stiffness, remains relatively limited. Given the critical role of early and sustained rehabilitation in maintaining surgical gains and preventing recontracture, it is essential to evaluate whether DT systems can effectively replace traditional rehabilitation methods for elbow contracture. This study seeks to fill the current gap in the literature by rigorously comparing DT systems with conventional outpatient clinic-based training in patients following arthroscopic release for posttraumatic elbow stiffness.

The purpose of this study was to determine whether a DT system is noninferior to conventional outpatient clinic-based training in improving elbow ROM and other functional outcomes after arthroscopic release for posttraumatic elbow stiffness.

## Methods

### Study Setting and Design

This study was designed as a noninferiority randomized controlled trial with a 1:1 allocation ratio and was conducted at a single center, the Rehabilitation Department of Shanghai Sixth People’s Hospital, from September 2020 to June 2024. The trial aimed to compare the efficacy of a DT system with that of conventional outpatient clinic-based training for patients undergoing arthroscopic release for posttraumatic elbow stiffness. This study was reported in accordance with the CONSORT (Consolidated Standards of Reporting Trials) extension for noninferiority and equivalence trials (CONSORT-NI), and the completed CONSORT checklist is provided in Multimedia Appendix 1.

### Ethical Considerations

The study received ethical approval from the Ethics Committee of Shanghai Sixth People’s Hospital (institutional review board approval number 2020-KY-089[K]) and was conducted in strict accordance with the principles outlined in the Declaration of Helsinki. All participants provided written informed consent before enrollment in the study. In addition, the study protocol was officially registered with the Chinese Clinical Trial Registry (identifier Chictr2400093415). To ensure privacy and confidentiality, all collected data were coded (deidentified) and securely stored using the hospital’s electronic data capture system on local servers accessible only to the research team. No financial compensation or other incentives were provided to participants for their involvement in this study.

### Participants

Patients were recruited from the Department of Orthopedics at Shanghai Sixth People’s Hospital between September 2020 and June 2024. The recruitment process involved 2 complementary approaches. First, orthopedic surgeons identified potentially eligible patients during routine preoperative evaluations and referred them to the research team. Second, the research team reviewed the weekly elective surgical schedule to identify patients scheduled for arthroscopic release of posttraumatic elbow contracture. All potentially eligible patients were approached by a member of the research team (YW), who explained the study objectives and procedures in detail. Those who expressed interest were formally screened using the predefined inclusion and exclusion criteria. Informed consent was obtained from patients who met the eligibility criteria and agreed to participate. Baseline data were collected before randomization. Participants were then consecutively allocated to either the DT system group or the conventional outpatient clinic-based training group (CT group) using a concealed randomization method. The assigned training protocol was initiated within the first postoperative week. The inclusion and exclusion criteria are listed in Textbox 1.

**Inclusion and exclusion criteria.**

**1. Inclusion criteria**

- Patients who are 16 years of age or older.
- Patients who have a lack of elbow flexion or extension causing functional impairment that has been present for at least 6 months.
- Patients who have failed to respond to nonoperative treatment.
- Patients who are scheduled to undergo secondary arthroscopic capsulectomy or osteocapsular arthroplasty.

**2. Exclusion criteria**

- Patients with contraindications for rehabilitation or regional brachial plexus block use, such as those with bleeding diathesis, taking anticoagulants, or experiencing severe shoulder range of motion limitations.
- Patients with progressive or persistent neuropathy or neuritis.
- Those with preexisting conditions that might hinder their ability to fully participate in rehabilitation, including neuromuscular or psychosocial conditions.
- Patients with ongoing or recurrent contracture due to inflammatory diseases such as rheumatoid arthritis, juvenile idiopathic arthritis, or chondrolysis.
- Patients presenting with elbow joint infections or a history of previous joint infections.
- Patients with structural anomalies that could restrict elbow motion, unrelated to the condition being treated, such as dysplasia, malunion, osteonecrosis, or congenital deformities.
- Cases where a reasonable restoration of motion and function was not anticipated.
- Patients with insufficient postoperative regional anesthesia.
- Those experiencing intraoperative or postoperative complications that might impact the study’s outcomes.
- Patients dealing with injuries or diseases during the postoperative period that could affect elbow function.
- Patients for whom arranging postoperative physical therapy appointments was not feasible.
- Cases where a significant portion of the procedure was performed in an open surgical manner.

### Sample Size Calculation

A sample size of 73 patients was estimated to provide 80% power (2-sided α=.025) to detect a 10° difference in ROM at 12 weeks postoperatively. The 10° noninferiority margin was prespecified in the registered protocol and selected based on multiple lines of evidence. Sun et al [10] reported a distribution-based minimal clinically important difference (MCID) of 14.1° and an anchor-based MCID of 25.0° for the total elbow flexion-extension arc after arthrolysis, with a minimal detectable change at the 90% confidence level of 10.5°. In addition, O’Driscoll et al [11] observed a clinically meaningful between-group ROM difference of 9° in a trial comparing rehabilitation strategies after arthroscopic elbow contracture release. Our 10° margin was therefore more conservative than the published MCID estimates and approximated the MDC_90_ threshold, ensuring that any clinically important difference would be detected. To mitigate potential attrition, we increased the target enrollment to 122 patients, assuming a 40% dropout rate.

### Intervention and Control Group

#### DT Group

Patients assigned to the DT group received individually tailored PEST through a mobile app (Joymotion R software; Shanghai Medmotion Medical Management Co, Ltd). On the day of discharge, a technician installed the app on each patient’s mobile device, and patients used their home Wi-Fi to access the program. The app provided individually tailored PEST with detailed instructions, recorded exercise completion rates, and offered real-time feedback on training performance. In addition, it enabled 2-way video and audio interactions with a physiotherapist (PT) at the rehabilitation center. Each week, the supervising PT initiated a scheduled conference with the patient to monitor progress, adjust the exercise regimen, and address any concerns. The PEST was individually prescribed by the supervising PT via the DT system and assigned as daily tasks to ensure continuity and adherence throughout the 12-week postoperative period.

#### CT Group

Patients in the control group received conventional outpatient clinic-based training (3-4 times per week), which consisted of scheduled training sessions and on-site PT supervision. This traditional approach involved direct, hands-on guidance and regular monitoring during therapy sessions at the Rehabilitation Department of Shanghai Sixth People’s Hospital. Unlike the DT group, the control group did not use a mobile app or remote monitoring tools, and their rehabilitation was delivered entirely in a clinical setting.

Both groups followed a standardized 12-week PEST protocol (detailed in Multimedia Appendix 2). During weeks 1 and 2, patients performed pain-free passive and active-assisted ROM exercises hourly, along with grade I-III joint mobilizations and cryotherapy. Isometric strengthening was initiated at week 2. During weeks 3 and 4, light isotonic resistance exercises were introduced and progressed based on the achievement of near-functional ROM and tolerable pain (Visual Analog Scale score ≤4). During weeks 5-8, moderate-to-advanced resistance, eccentric loading, and sport- or work-specific functional training were added. During weeks 9-12, strengthening was maximized, and patients transitioned to self-managed maintenance programs. All exercises were performed within a pain-limited range; no absolute ROM ceilings were enforced. Outcome assessments were conducted at predetermined intervals to compare improvements in elbow function, ROM, and overall recovery.

### Outcome Measures

#### Overview

Functional outcome measures were collected by physical therapists in outpatient clinics. Self-assessed outcome measures were also completed in the outpatient clinic using online questionnaires, with assistance from 2 physical therapists who were blinded to group allocation. Follow-up assessments were conducted at 4, 12, and 24 weeks after surgery.

#### Primary Outcome

The primary outcome was active elbow flexion-extension ROM (in degrees) measured at 12 weeks postoperatively. A standard handheld goniometer was used to measure the angle from full extension to maximum flexion of the affected elbow, providing an objective measure of motion.

#### Secondary Outcomes

Elbow flexion-extension ROM was measured in degrees using a goniometer at 4, 12, and 24 weeks after surgery. Forearm rotation ROM was also assessed at these same time points using a goniometer. All ROM measurements were performed with the patient’s arm in a standardized position to ensure consistency, and greater angles indicated improved elbow joint flexibility.

Elbow flexor strength was evaluated at postoperative follow-up visits using a calibrated Baltimore Therapeutic Equipment (BTE) work simulator. Isometric strength was measured as the maximal voluntary contraction of the elbow flexor muscles maintained for 5 seconds with the elbow at a fixed angle. Dynamic strength was measured by having the patient perform repeated elbow flexion movements against a preset resistance on the BTE machine, simulating a functional lifting motion. Peak force or torque values obtained during each test were recorded. For standardization, strength outcomes were expressed as a percentage of the strength of the patient’s unaffected contralateral elbow, allowing direct comparison with the healthy side (see Multimedia Appendix 2).

The American Shoulder and Elbow Surgeons (ASES) Shoulder Score Elbow Outcome questionnaire was administered at 4, 12, and 24 weeks to assess elbow function and pain from the patient’s perspective. It yields 2 subcomponents: a pain score (typically assessed using a visual analog scale or pain rating, with 0 indicating no pain and higher values indicating worse pain) and a function score (based on the patient’s ability to perform daily activities requiring elbow use). Each subscore contributes to an overall ASES elbow score (range 0-100 points), with higher scores indicating better function and less pain. For this study, the pain and function subscores were analyzed separately to assess improvements in each recovery domain.

Upper extremity disability and symptoms were evaluated using the Disabilities of the Arm, Shoulder, and Hand (DASH) questionnaire at 4, 12, and 24 weeks postoperatively. The DASH is a 30-item patient-reported outcome measure in which patients rate the difficulty of and interference with performing various physical activities involving the arm, shoulder, or hand. Scores range from 0 to 100, with 0 reflecting no disability and 100 indicating the most severe disability [12]. A decrease in the DASH score over time indicates improvement in arm function and reduction in disability [11].

General health-related quality of life was assessed at 4, 12, and 24 weeks using the EQ-5D-5L questionnaire. The EQ-5D-5L is a standardized instrument that covers 5 dimensions of health (mobility, self-care, usual activities, pain/discomfort, and anxiety/depression), each rated on 5 levels of severity [13]. Patients completed the questionnaire, and the responses were converted into a health utility index value and an EQ-5D Visual Analog Scale score for overall self-rated health. This provided a broad measure of patients’ quality of life and well-being during recovery, with improvements in EQ-5D-5L scores indicating better overall health status as rehabilitation progressed.

### Randomization, Allocation, and Blinding

A computer-generated randomization sequence was used to assign participants to either the DT or CT group. Block randomization with varying block sizes ensured balanced allocation over time. Allocation concealment was maintained using sealed, opaque, sequentially numbered envelopes prepared by an independent researcher (SY) who was not involved in patient recruitment or assessment. Following the initial assessment, a volunteer PT distributed the envelopes according to the randomization sequence. These consecutively numbered, sealed, opaque envelopes were stored in a secure location accessible only to the unblinded lead researcher-PT (SL). This PT then scheduled the participants’ postoperative training program and introduced the DT system.

Because of the nature of the interventions, blinding of patients and treating physical therapists was not feasible. However, outcome assessors responsible for measuring ROM and strength and administering standardized questionnaires (DASH, EQ-5D-5L, and ASES) were blinded to group allocation. Data analysts were also blinded to treatment assignments during statistical analysis to minimize potential bias. To support blinding during data collection, precoded data forms were used so that intervention group identity remained masked during data entry and evaluation.

### Statistical Analysis

Patient data were securely coded and stored using the hospital’s electronic data capture system on local servers. Baseline characteristics were summarized as mean (SD) for continuous variables and as frequencies and percentages for categorical variables. Between-group differences in baseline characteristics were assessed using independent 2-sample *t* tests (unpaired and 2-tailed) for continuous variables and chi-square tests for categorical variables. All hypothesis tests were 2-tailed, and a *P* value <.05 was considered statistically significant for these comparisons.

The primary efficacy analysis was performed on the intention-to-treat population in accordance with noninferiority trial guidelines. Cost-effectiveness and adverse event analyses were also conducted using the intention-to-treat population, with all randomized patients analyzed in their originally assigned groups.

The primary outcome was elbow flexion-extension ROM at 12 weeks postoperatively. Primary and secondary outcomes were analyzed using linear mixed-effects models to account for repeated-measures data collected over time. Fixed effects included group (DT vs CT), time (baseline, 4 weeks, 12 weeks, and 24 weeks), and their interaction (group × time), whereas age, sex, and the baseline value of each respective outcome were included as covariates. Specifically, for each outcome variable, the corresponding baseline measurement was entered as a covariate in the model, consistent with standard recommendations for controlling regression to the mean in randomized trials. Random effects were specified to account for within-participant variability, and least-squares means with 95% CIs were calculated for group comparisons at each time point. Noninferiority was established if the upper bound of the 95% CI for the between-group difference in 12-week ROM did not exceed 10°, indicating that the DT group was not more than 10° worse than the CT group. To account for rounding in range-of-motion measurements, we applied a *P* value correction method following the recommendation of Zdravkovic and Jost [14], setting the significance threshold at .03 for outcomes assessing mean differences in ROM. For all other outcomes, a *P* value <.05 was considered statistically significant.

Continuous secondary outcomes were also analyzed using linear mixed-effects models similar to those used for the primary outcome analysis, with adjustment for baseline values when available. Dichotomous outcomes were compared using Cochran-Mantel-Haenszel chi-square tests, controlling for baseline contracture severity.

For the multiple secondary end points, both the Bonferroni correction and the Holm step-down procedure were applied to adjust for multiple comparisons. Seven secondary efficacy outcomes were assessed at the 12-week primary end point: forearm rotation, isometric strength, dynamic strength, ASES function, ASES pain, DASH, and EQ-5D index. The Bonferroni correction set the significance threshold for each secondary outcome at α=.05/7. As the secondary outcomes were positively correlated, the standard Bonferroni approach was considered overly conservative. Therefore, the Holm step-down procedure was also applied because it controls the familywise error rate identically to the Bonferroni method while offering uniformly greater statistical power. Both methods yielded consistent conclusions. *P* values for secondary outcomes were interpreted against both adjusted thresholds. The primary outcome (noninferiority in ROM) was analyzed using a 1-sided alpha of .025 and was not subject to multiplicity adjustment.

The incremental cost-effectiveness ratio (ICER) was used to assess the cost-effectiveness of the DT group compared with the CT group. The ICER represents the difference in costs and outcomes between the groups. The numerator of the cost-effectiveness ratio was calculated as the monetary cost of the DT intervention minus the monetary cost of CT. Annual project costs were estimated by extrapolating the 12-week implementation costs. The denominator was calculated as the ROM gained in the DT group minus the ROM gained in the CT group at 12 weeks. Bootstrapping was used for pairwise comparisons of mean costs and effects between the DT and CT groups. CIs for mean differences in effects were obtained using bootstrapping with 1000 replications. The bootstrapped cost-effect pairs were also graphically represented on a cost-effectiveness plane [15].

Missing data in this study occurred exclusively at the visit level. If a patient missed a follow-up visit, all outcomes from that visit were missing; however, data from attended visits remained available. There was no item-level missingness within completed visits. Given this structure, mixed-effects models in the intention-to-treat analysis retained all available data for each participant. No imputation was applied to address missing values. As data loss occurred at the level of entire visits rather than within-visit fields, tests for data missing completely at random, such as Little missing completely at random test, were not performed. The analysis was repeated in the per-protocol population, which included only patients who completed all scheduled follow-up assessments. Missing outcome data were therefore addressed using both intention-to-treat and per-protocol analytical strategies.

## Results

### Participants

**Figure 1:**
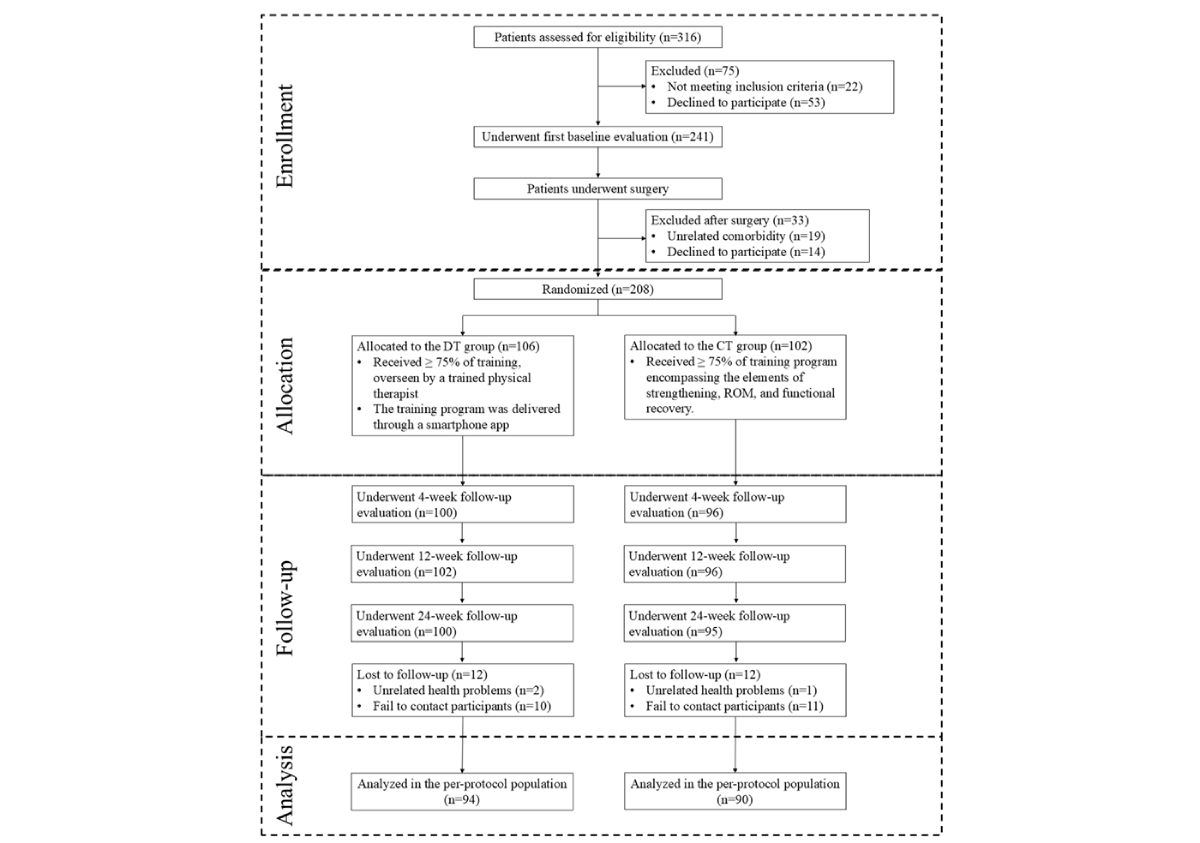
Flowchart of the study from enrollment to analysis. CT: conventional training; DT: digital training; ROM: range of motion.

The DT group (n=106) and the CT group (n=102) were well matched at baseline (Table 1). The CONSORT flowchart for both the control and intervention groups, detailing exclusions and follow-up losses, is illustrated in Figure 1. The mean age was 47 years in the DT group and 48 years in the CT group, with males comprising 77 of 106 (72.6%) patients and 80 of 102 (78.4%) patients, respectively. The mean BMI was approximately 23 kg/m^2^ in the DT group and 22 kg/m^2^ in the CT group. Manual workers accounted for 59 of 106 (55.7%) patients in the DT group and 60 of 102 (58.8%) patients in the CT group, whereas 73 of 106 (68.9%) patients and 56 of 102 (54.9%) patients, respectively, had achieved at least a high school education. Most patients were covered by government insurance. The numbers of current smokers (*P*=.39), alcohol users (*P*=.28), and users of paracetamol and nonsteroidal anti-inflammatory drugs (*P*=.55) showed no significant between-group differences. Clinically, most patients had no prior history of significant elbow injuries, such as distal humerus, capitellum, olecranon, Monteggia, or radial head fractures, and only a small proportion had previously undergone surgical treatment for these conditions. No significant between-group differences were observed in records of previous operations (osteocapsular arthroplasty, *P*=.14; capsular release, *P*=.14; ulnar nerve management—limited decompression, *P*=.76; ulnar nerve management—subcutaneous transposition, *P*=.76; removal of heterotopic ossification, *P*=.61; radial head excision, *P*=.94; and hardware removal, *P*=.15). Baseline elbow function was comparable between groups, with mean flexion-extension ROMs of approximately 78°-79°, forearm rotation ROMs ranging from 76° to 91°, and isometric and dynamic flexion strength values of approximately 69%-70% and 77% of the contralateral side, respectively. Patient-reported outcomes, including ASES scores, DASH scores, and EQ-5D-5L indices, were also similar, reflecting moderate impairment and reduced quality of life in both groups.

**Table 1:** Baseline characteristics of the DT^a^ and CT^b^ groups (intention-to-treat population).

Characteristic				DT group (n=106)		CT group (n=102)		*P* value	
Demographic characteristics									
	Male patients, n (%)			69 (65.1)		73 (71.6)		.32	
	Age (years), mean (SD)			47 (7.30)		48 (7.18)		.25	
	BMI (kg/m^2^), mean (SD)			23 (2.86)		22 (3.21)		.19	
	Occupation, n (%)								.64
		Manual worker	59 (55.7)		60 (58.8)				
		Nonmanual worker	47 (44.3)		42 (41.2)				
	Education level, n (%)								.04
		Lower than high school	33 (31.1)		46 (45.1)				
		Equal/higher to high school	73 (68.9)		56 (54.9)				
	Insurance type, n (%)								.70
		Government	69 (65.1)		70 (68.6)				
		Commercial	10 (9.4)		11 (10.8)				
		Self-financed	27 (25.5)		21 (20.6)				
	Current smoker, n (%)			51 (48.1)		43 (42.2)		.39	
	Current alcohol use, n (%)			43 (40.6)		34 (33.3)		.28	
	Paracetamol and nonsteroidal anti-inflammatory drug, n (%)			32 (30.2)		27 (26.5)		.55	
History of previous etiology for the elbow									
	Distal humerus fracture, n (%)								.33
		None	86 (81.1)		81 (79.4)				
		Conservative treatment	14 (13.2)		10 (9.8)				
		Surgical treatment	6 (5.7)		11 (10.8)				
	Capitellum fracture, n (%)								.84
		None	83 (78.3)		81 (79.4)				
		Conservative treatment	14 (13.2)		11 (10.8)				
		Surgical treatment	9 (8.5)		10 (9.8)				
	Olecranon fracture, n (%)								.99
		None	86 (81.1)		83 (81.4)				
		Conservative treatment	16 (15.1)		15 (14.7)				
		Surgical treatment	4 (3.8)		4 (3.9)				
	Monteggia fracture, n (%)								.41
		None	95 (89.6)		85 (83.3)				
		Conservative treatment	7 (6.6)		11 (10.8)				
		Surgical treatment	4 (3.8)		6 (5.9)				
	Radial head, n (%)								.35
		None	85 (80.2)		88 (86.3)				
		Conservative treatment	15 (14.2)		8 (7.8)				
		Surgical treatment	6 (5.7)		6 (5.9)				
	Terrible triad injury, n (%)								.89
		None	95 (89.6)		92 (90.2)				
		Conservative treatment	0 (0)		0 (0)				
		Surgical treatment	11 (10.4)		10 (9.8)				
Records of previous operation									
	Osteocapsular arthroplasty, n (%)			82 (77.4)		87 (85.3)		.14	
	Capsular release (soft tissue only), n (%)			24 (22.6)		15 (14.7)		.14	
	Ulnar nerve management—limited decompression, n (%)			92 (86.8)		87 (85.3)		.76	
	Ulnar nerve management—subcutaneous transposition, n (%)			14 (13.2)		15 (14.7)		.76	
	Removal of heterotopic ossification, n (%)			13 (12.3)		15 (14.7)		.61	
	Radial head excision with or without Interposition arthroplasty, n (%)			8 (7.5)		8 (7.8)		.94	
	Hardware removal, n (%)			13 (12.3)		20 (19.6)		.15	
	Tourniquet time (minutes), mean (SD)			90 (29.65)		89 (27.01)		.65	
Function, mean (SD)^c^									
	ROM^d^ of elbow flexion to extension motion (degrees)			78 (22.08)		79 (21.97)		.81	
	ROM of forearm rotation (degrees)			91 (44.06)		76 (42.47)		.01	
	Flexion strength—isometric elbow flexion strength (%)			69 (3.78)		70 (4.06)		.16	
	Flexion strength—dynamic elbow flexion strength (%)			77 (4.20)		77 (3.78)		.45	
PROMs^e^, mean (SD)									
	ASES^f^ Function subscore (points)			22 (2.92)		22 (2.70)		.39	
	ASES Pain subscore (points)			22 (3.13)		23 (2.69)		.76	
	DASH^g^ score (points)			40 (5.88)		40 (4.46)		.93	
	EQ-5D-5L			0.71 (0.05)		0.71 (0.04)		.87	

^a^DT: digital training.

^b^CT: conventional training.

^c^Isometric flexion strength and dynamic flexion strength were measured and compared with the contralateral side using a BTE machine (Simulator II).

^d^ROM: range of motion.

^e^PROM: passive range of motion.

^f^ASES: American Shoulder and Elbow Surgeons.

^g^DASH: Disabilities of the Arm, Shoulder, and Hand.

### Primary and Secondary Outcomes

Using the linear mixed-effects model, the adjusted between-group difference in 12-week elbow flexion-extension ROM was –1.6° (95% CI –8.2° to 4.9%; Tables 2 and 3), indicating noninferiority of DT compared with CT. A complete-case analysis excluding 12 patients without ROM follow-up yielded a similar difference of –1.5° (95% CI –8.8° to 5.0°), confirming that the primary outcome was not sensitive to the handling of missing data. Thus, the conclusion of noninferiority remained unchanged when only observed cases were analyzed compared with multiply imputed data.

Secondary outcomes improved comparably in both groups, with no between-group differences meeting the adjusted significance criteria after Bonferroni correction for multiple end points. Forearm rotation ROM increased slightly more in the DT group, with an adjusted difference of 14.2° (95% CI 2.9°-25.6°). Isometric and dynamic flexion strength gains were nearly identical between groups, with differences of 0.04% (95% CI –2.1% to 2.2%) and 0.26% (95% CI –1.5% to 2.0%), respectively, when normalized to the contralateral side. Functional recovery, assessed using the ASES function subscore, showed a small difference of 0.6 points (95% CI –0.3 to 1.5), indicating similar functional improvement in both groups. Pain reduction, measured using the ASES pain subscore, was comparable, with a near-zero adjusted difference (95% CI –8.3 to 8.5). The DASH score, reflecting disability, improved similarly in both groups, with a minimal between-group difference of 0.23 points (95% CI –1.54 to 1.99). Lastly, health-related quality of life, measured using the EQ-5D-5L, showed no meaningful between-group difference, with an adjusted index difference of 0.001 (95% CI –0.012 to 0.015; Tables 2 and 3). Thus, forearm rotation (*P*<.001), ASES function (*P*<.001), and EQ-5D-5L (*P*=.003) showed statistically significant between-group differences, whereas isometric strength (*P*=.76), dynamic strength (*P*=.67), ASES pain (*P*=.18), and DASH (*P*=.87) showed no significant between-group differences. Sensitivity analyses using the per-protocol population showed similar results (Multimedia Appendices 3 and 4). Sensitivity analyses of secondary outcomes at 12 weeks using Bonferroni and Holm step-down adjustments for multiple comparisons are presented in Multimedia Appendix 5. An additional sensitivity analysis that included education level as a covariate in the primary mixed-effects model yielded an adjusted between-group difference of –1.7° (95% CI –8.4° to 5.0°), confirming that the noninferiority conclusion was robust to adjustment for this baseline imbalance.

**Table 2:** Unadjusted changes in outcomes for the DT^a^ and CT^b^ groups at weeks 4, 12, and 24 after surgery (intention-to-treat population).^c^

Outcome^d^	4 weeks after surgery				12 weeks after surgery				24 weeks after surgery		
	DT group (n=106), mean (SD)	CT group (n=102), mean (SD)	*P* value	DT group (n=106), mean (SD)		CT group (n=102), mean (SD)	*P* value	DT group (n=106), mean (SD)		CT group (n=102), mean (SD)	*P* value
ROM^e^ of elbow flexion to extension motion (degrees)	9.36 (12.58)	10.17 (14.77)	.69	19.47 (15.30)		20.89 (17.38)	.55	22.82 (15.13)		24.22 (16.61)	.55
ROM of forearm rotation (degrees)	25.96 (20.49)	26.39 (20.29)	.89	43.24 (25.07)		46.67 (22.02)	.33	52.07 (26.64)		56.44 (23.96)	.24
Flexion strength—isometric elbow flexion strength (%)	5.27 (6.10)	5.01 (6.10)	.77	21.53 (6.04)		20.41 (6.09)	.21	25.93 (5.35)		24.38 (5.40)	.05
Flexion strength—dynamic elbow flexion strength (%)	5.11 (5.87)	4.25 (4.79)	.28	17.01 (5.58)		16.53 (5.28)	.55	23.29 (5.42)		23.04 (5.38)	.75
ASES^f^ Function subscore (points)	4.31 (2.89)	3.98 (2.99)	.45	8.72 (3.89)		8.23 (3.95)	.39	6.45 (4.29)		5.93 (4.11)	.40
ASES Pain subscore (points)	–4.99 (4.41)	–5.67 (4.49)	.30	–12.72 (6.51)		–13.12 (6.70)	.68	–10.90 (6.55)		–11.26 (6.94)	.72
DASH^g^ score (points)	–11.97 (7.70)	–12.50 (7.09)	.63	–24.94 (9.36)		–25.49 (8.98)	.68	–26.18 (9.22)		–26.59 (8.77)	.76
EQ-5D-5L^h^	0.00 (0.00)	0.00 (0.00)	N/A^i^	0.08 (0.05)		0.08 (0.06)	.56	0.08 (0.05)		0.09 (0.06)	.56

^a^DT: digital training.

^b^CT: conventional training.

^c^Values represent the mean change from baseline for each group, with *P* values comparing between-group differences at each follow-up point. All outcome measures were unadjusted.

^d^Isometric flexion strength and dynamic flexion strength were measured and compared with the contralateral side using a Baltimore Therapeutic Equipment machine (Simulator II).

^e^ROM: range of motion.

^f^ASES: American Shoulder and Elbow Surgeons.

^g^DASH: Disabilities of the Arm, Shoulder, and Hand.

^h^The EQ-5D-5L change of 0.00 (SD 0.00) at 4 weeks in both groups reflects the limited responsiveness of this generic health utility instrument in the early postoperative period. At 4 weeks after elbow surgery, improvements in joint-specific function are typically offset by ongoing postoperative recovery burden, resulting in negligible net change in global health utility. This pattern is consistent with published studies reporting limited EQ-5D sensitivity to change in upper extremity orthopedic populations during the early recovery phase.

^i^N/A: not applicable.

**Table 3:** Adjusted effectiveness estimates from linear mixed-effects models (intention-to-treat population).^a^

Outcome^b^	4 weeks after surgery				12 weeks after surgery				24 weeks after surgery		
	Coefficient	95% CI	*P* value	Coefficient		95% CI	*P* value	Coefficient		95% CI	*P* value
ROM^c^ of elbow flexion to extension motion (degrees)	–2.131	–8.843 to 4.580	.53	–2.810		–5.311 to –0.309	.03	–2.764		–4.592 to –0.934	.003
ROM of forearm rotation (degrees)	14.577	3.587 to 25.568	.009	13.317		10.422 to 16.213	<.001	12.942		10.540 to 15.345	<001
Flexion strength—isometric elbow flexion strength (%)	–0.356	–1.011 to 0.298	.29	–0.073		–0.539 to 0.393	.76	0.237		–0.392 to 0.867	.46
Flexion strength—dynamic elbow flexion strength (%)	–0.005	–0.751 to 0.742	.99	0.136		–0.487 to 0.759	.67	0.139		–0.283 to 0.561	.52
ASES^d^ Function subscore (points)	0.476	–0.426 to 1.377	.30	0.644		0.327 to 0.960	<.001	0.804		0.420 to 1.188	<.001
ASES Pain subscore (points)	0.255	–0.642 to 1.151	.58	0.298		–0.133 to 0.729	.17	0.317		–0.031 to 0.664	.07
DASH^e^ score (points)	–0.040	–1.669 to 1.589	.96	0.025		–0.270 to 0.320	.87	–0.017		–0.331 to 0.298	.92
EQ-5D-5L	–0.003	–0.0134 to 0.009	.65	–0.003		–0.006 to –0.001	.003	–0.005		–0.009 to –0.001	.007

^a^Each coefficient represents the estimated between-group difference in the change from baseline (intervention group minus control group) for the specified outcome at that follow-up time point. Positive coefficients indicate higher scores in the intervention group compared with the control group, whereas negative values indicate lower scores in the intervention group. Each estimate is presented with its 95% CI and corresponding *P* value. All outcome measures were adjusted for baseline values in the model.

^b^Isometric flexion strength and dynamic flexion strength were measured and compared with the contralateral side using a BTE machine (Simulator II).

^c^ROM: range of motion.

^d^ASES: American Shoulder and Elbow Surgeons.

^e^DASH: Disabilities of the Arm, Shoulder, and Hand.

### Cost-Effectiveness Outcomes

**Figure 2:**
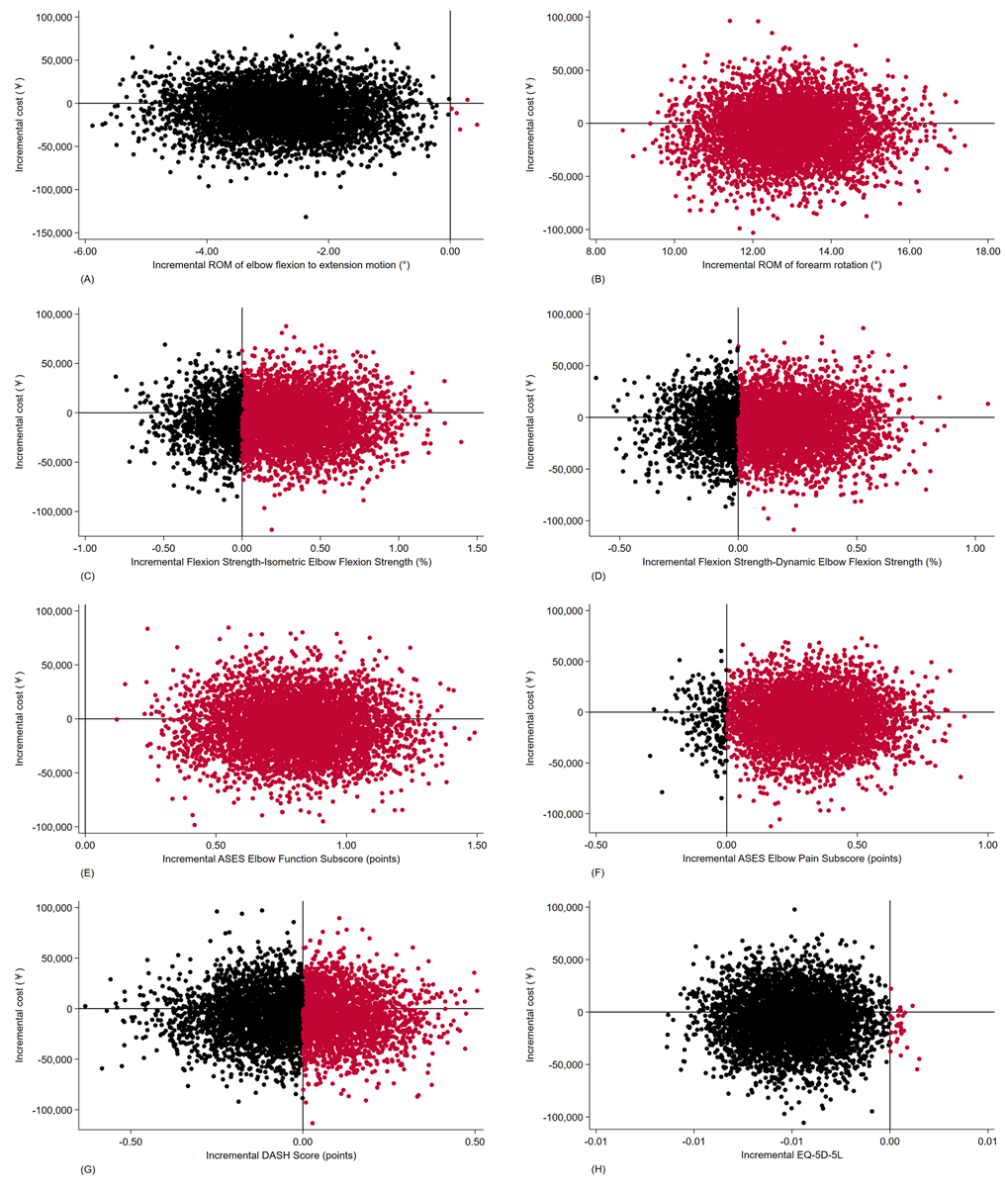
Bootstrapped cost-effectiveness planes (BCEPs) for the comparison of digital training (DT) vs conventional training (CT). Panels show the BCEPs for incremental outcomes of (A) elbow flexion-extension range of motion (ROM), (B) forearm rotation ROM, (C) isometric elbow flexion strength, (D) dynamic elbow flexion strength, (E) American Shoulder and Elbow Surgeons (ASES) function score, (F) ASES pain score, (G) Disabilities of the Arm, Shoulder, and Hand (DASH) score, and (H) EQ-5D-5L score. Each point represents an incremental cost-effect pair (ΔEffect on the horizontal axis and ΔCost on the vertical axis) derived from 1000 bootstrap replicates of the trial data. The origin (0,0) indicates no difference between groups. Points below the horizontal axis indicate that DT is less costly than CT, whereas points to the right of the vertical axis indicate that DT is more effective than CT. The cost-effectiveness acceptability curve illustrates the probability that DT is cost-effective compared with CT across a range of willingness-to-pay thresholds per unit of effect gained (per quality-adjusted life-year gained). The curve was generated from the same 1000 bootstrap replicates by calculating the proportion of simulations in which the net monetary benefit of DT compared with CT was positive at each willingness-to-pay threshold.

**Figure 3:**
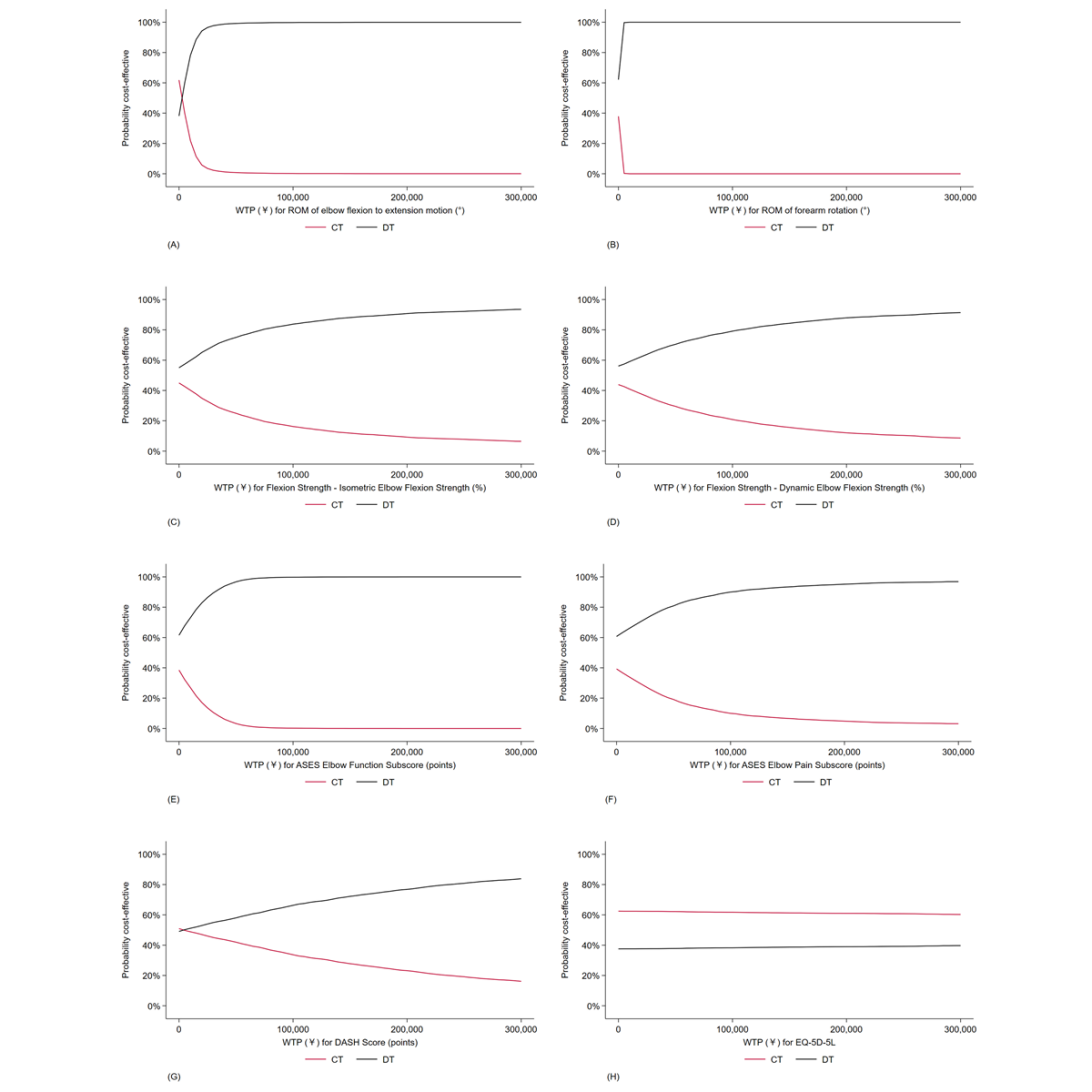
Cost-effectiveness acceptability curves (CEACs) for the comparison of digital training (DT) vs conventional training (CT). Panels show the CEACs for (A) elbow flexion-extension range of motion (ROM), (B) forearm rotation ROM, (C) isometric elbow flexion strength, (D) dynamic elbow flexion strength, (E) American Shoulder and Elbow Surgeons (ASES) function score, (F) ASES pain score, (G) Disabilities of the Arm, Shoulder, and Hand (DASH) score, and (H) EQ-5D-5L score. Each curve illustrates the probability that DT is cost-effective compared with CT across a range of willingness-to-pay (WTP) thresholds per unit of effect gained (per quality-adjusted life-year gained). The curves were derived from 1000 bootstrap replicates by calculating the proportion of simulations in which the net monetary benefit of DT compared with CT was positive at each WTP threshold.

The economic evaluation is presented as a short-term cost-consequence analysis given the limited 12-24-week time horizon, with bootstrapped results presented in Tables 4 and 5 and Figures 2 and 3. We note that the incremental health effects were very close to 0 for most outcomes, rendering formal cost-per-quality-adjusted life year ratios unstable and difficult to interpret. The results should therefore be interpreted as descriptive cost-consequence findings rather than as a definitive cost-utility evaluation. Figure 2 illustrates the incremental cost-effectiveness plane. Across 1000 bootstrap resamples, the incremental cost of DT versus CT was almost always negative (median CNY –7400 [US $–1087]), whereas the incremental effects clustered near 0. The majority of bootstrap iterations fell within the southeast quadrant (negative Δcost, positive Δeffect), indicating that DT was cost-saving and at least as effective as CT in most resamples. A minority of points fell within the southwest quadrant (cost-saving but slightly less effective), and virtually none fell within the costly quadrants, underscoring the consistency of the cost-saving result. Figure 3 shows the cost-effectiveness acceptability curve. At a willingness-to-pay threshold of CNY 0 (US $0) per unit of effect, the probability that DT was cost-effective was 75%. This probability increased to 78% at a threshold of CNY 134,000 (US $19,677) per quality-adjusted life year and approached 80% as willingness-to-pay increased. These probabilistic findings suggest that DT was associated with lower costs in most bootstrap resamples. However, because incremental health effects clustered near 0, the cost-effectiveness acceptability curve should be interpreted with caution. The high probability of cost-effectiveness primarily reflects the cost-saving nature of the digital intervention rather than a meaningful differential in health effects. These short-term findings should not be extrapolated to longer time horizons without further study because downstream costs or savings associated with preventing recontracture or reoperation were not captured.

**Table 4:** Average total cost per patient in the DT^a^ and CT^b^ groups during the 24 weeks after the surgery (intention-to-treat population).^c^

Cost category (CNY^d^)		DT group (n=106), mean (SD)	CT group (n=102), mean (SD)	*P* value
Direct medical cost				
	Physical therapist cost	13,960.00 (0.00)	13,706.88 (2036.32)	.21
	Hospital stay cost	7001.43 (1020.29)	6984.06 (908.39)	.90
	Primary care cost	249.00 (34.80)	2438.95 (362.33)	<.001
	Secondary care cost	5984.12 (1005.90)	7922.16 (1227.93)	<.001
Direct nonmedical cost				
	Paid home cost	1211.67 (323.50)	1449.24 (347.61)	<.001
	Medication	8057.68 (860.30)	8668.13 (1531.21)	.001
	Transportation cost	775.05 (162.58)	2025.34 (335.97)	<.001
	Nutrition cost	4569.25 (513.63)	5138.81 (493.07)	<.001
Indirect cost				
	Lost wages for patients	31,323.61 (16,406.72)	32,576.12 (16977.68)	.60
	Lost wages for families	4218.69 (4209.30)	3859.40 (4576.58)	.57
Total cost		77,350.51 (16,923.96)	84,769.09 (17935.57)	.003

^a^DT: digital training.

^b^CT: conventional training.

^c^All relevant costs were captured from a societal perspective. This included direct medical costs (hospital stay, primary and secondary care, rehabilitation and physical therapy costs, and medication costs), direct nonmedical costs related to care (transportation for medical visits and any specialized nutritional support during recovery), and indirect costs due to productivity loss (lost wages for patients during recovery or disability, and lost income for family members/caregivers, if applicable). Costs were obtained from hospital billing records and patient self-reports where needed (wage losses). All costs are reported in 2024 Chinese Yuan, as 2024 was the end of the enrollment period; costs incurred in earlier years were adjusted to 2024 price levels using the consumer price index. Costs and outcomes occurring beyond the initial year were discounted at an annual rate of 3% to reflect time preference, consistent with standard practice in health economic evaluations.

^d^CNY 1=US $0.15.

**Table 5:** ICER^a^ (intention-to-treat population).^b,c^

Main analysis—mixed effects	Values
Incremental cost (CNY^d^)	–7418.58
Incremental ROM^e^ of elbow flexion to extension motion (degrees)	–1.663 (–8.286 to 4.961)
Incremental ROM of forearm rotation (degrees)	13.07 (2.110 to 24.031)
Incremental flexion strength—isometric elbow flexion strength (%)	0.23 (–2.163 to 2.624)
Incremental flexion strength—dynamic elbow flexion strength (%)	0.159 (–2.079 to 2.397)
Incremental ASES^f^ Elbow Function subscore (points)	0.631 (–0.280 to 1.541)
Incremental ASES Elbow Pain subscore (points)	0.032 (–1.609 to 1.673)
Incremental DASH^g^ score (points)	0.253 (–1.639 to 2.145)
Incremental EQ-5D-5L	0.000 (–0.015 to 0.015)
ICER ROM of elbow flexion to extension motion (degrees)	4460.96
ICER ROM of forearm rotation (degrees)	–567.60
ICER flexion strength—isometric elbow flexion strength (%)	–32,254.70
ICER flexion strength—dynamic elbow flexion strength (%)	–46,657.74
ICER ASES Function subscore (points)	–11,756.86
ICER ASES Pain subscore (points)	–231,830.63
ICER DASH score (points)	–29,322.45
ICER EQ-5D-5L	–112,402,727.27

^a^ICER: incremental cost-effectiveness ratio.

^b^The table displays the incremental cost, incremental effect in primary outcome scores, and the resulting ICER. ICER is defined as the incremental cost divided by the incremental effect, representing the additional cost per unit improvement in outcome for the intervention group versus the control group. Incremental effects are given for all outcome measures. ICER values are presented for the 12-month follow-up under both the intention-to-treat and per-protocol analyses. A negative ICER indicates that the intervention group achieved equivalent or better outcomes at a lower cost compared with the control group.

^c^Values are reported as follows: incremental effects as coefficient (95% CI), and ICER values as point estimates computed from the ratio of incremental cost to incremental effect.

^d^CNY 1=US $0.15.

^e^ROM: range of motion.

^f^ASES: American Shoulder and Elbow Surgeons.

^g^DASH: Disabilities of the Arm, Shoulder, and Hand.

### Adherence and Acceptability

Patients in both groups attended approximately 3.4-3.5 sessions per week. The DT group rated agreement with the assigned exercise plan at 8.2, compared with 8.4 in the CT group. Adherence to the recommended exercise program was high in both groups, with scores of 8.5 and 8.6, respectively. Similarly, ratings for pain relief were 8.7 versus 8.8, and ratings for functional improvement were 8.8 versus 8.9. Overall satisfaction with the exercise protocol was very high, at 9.5 in the DT group and 9.6 in the CT group. No significant between-group differences were observed, indicating excellent adherence and acceptability (Table 6).

**Table 6:** Patients’ adherence to treatment.^a^

Outcome measure			DT^b^ group (n=106)		CT^c^ group (n=102)	*P* value
Number of sessions performed per week, mean (SD)			3.4 (0.9)		3.5 (1.0)	.43
Agreement with the following questions (0-10)^d^, mean (SD)						
	To what extent did you agree to accept the allocated exercise plan?	8.2 (1.3)		8.4 (1.2)		.28
	To what extent did you do the exercise program as recommended?	8.5 (1.4)		8.6 (1.4)		.44
	To what extent do you agree that the intervention relieved your pain?	8.7 (1.4)		8.8 (1.3)		.63
	To what extent do you agree that the intervention improved your function?	8.8 (1.1)		8.9 (1.2)		.82
	To what extent were you satisfied with the exercise protocol?	9.5 (0.5)		9.6 (0.4)		.11

^a^Adherence data were retrieved from patients’ logbooks and the health information system in both groups.

^b^DT: digital training.

^c^CT: conventional training.

^d^0=strongly disagree to 10=strongly agree.

### Adverse Events

Adverse events occurred in 29 of 106 (27.4%) patients in the DT group and 32 of 102 (31.4%) patients in the CT group. Therapy-related events occurred in 4 patients in the DT group and 6 patients in the CT group, with common issues including pain, swelling, and tenosynovitis. Serious adverse events leading to study withdrawal occurred in 2 (2%) DT patients and 1 (1%) CT patient (Table 7).

**Table 7:** Adverse events and serious adverse events.

Adverse events		DT^a^ group (n=106)	CT^b^ group (n=102)
Patients with adverse events, n (%)		29 (27.4)	32 (31.4)
Events related to study therapy, n		4	6
Events unrelated to study therapy, n		36	41
Type of event, n			
	Pain	11	14
	Swelling	7	8
	Muscle strain	1	2
	Tenosynovitis	3	2
	Signs of infection (swelling, redness, heat, or pus)	0	1^c^
	Mobilization under anesthesia	1	1
Other, n			
	Nausea and dizziness	0	2
	Shoulder pain	2	3
	Wrist pain	4	2
	Anxiety about elbow recovery	7	6
Serious adverse events^d^			
	Patients with serious adverse events, n (%)	2 (1.9)	1 (1.0)
	Events related to study therapy, n	0	0
Events unrelated to study therapy, n		2	1
	Hip fracture due to fall	1^e^	0
	Waist fracture due to fall	1^e^	0
	Spinal surgery	0	1^e^

^a^DT: digital training system.

^b^CT: conventional outpatient clinic-based training.

^c^One patient sustained a mild infection of the surgical site and healed with conservative treatment.

^d^Patients with serious adverse events were automatically withdrawn from the study.

^e^Two patients in the DT group sustained accidental falls during daily activities unrelated to the rehabilitation protocol. One patient sustained a hip fracture while walking outdoors (approximately 6 weeks postoperatively, not during or immediately after an exercise session) and underwent surgical fixation. Another patient sustained a lumbar vertebral fracture from a fall at home during routine household activities (approximately 8 weeks postoperatively) and was treated conservatively. Neither event was temporally associated with prescribed rehabilitation exercises, and the DT protocol did not include balance-challenging or weight-bearing lower extremity exercises. Both events were adjudicated as unrelated to the study intervention. Both patients were automatically withdrawn from the study.

## Discussion

### Principal Findings

This trial demonstrates that a DT system in which patients receive individually tailored PEST yields outcomes comparable to those of conventional outpatient clinic-based training after arthroscopic release for posttraumatic elbow stiffness. At 12 weeks, the DT group met the prespecified noninferiority criterion for elbow flexion-extension ROM relative to the CT group. The between-group difference was –1.6° (95% CI –8.2° to 4.9°), with the upper bound of the CI well within the 10° noninferiority margin. However, the lower bound of –8.2° approaches a magnitude that some clinicians may consider clinically relevant in a population with stiff elbows. Statistical noninferiority does not necessarily imply clinical interchangeability, and individual patient factors should inform treatment selection. Secondary outcomes, including pain relief and functional improvement, were also comparable between groups. Both interventions demonstrated high adherence, and no serious adverse events related to treatment were observed. The inclusion of bootstrap-based uncertainty analyses provides additional support for the economic advantage of the digital program. The scatter distribution on the cost-effectiveness plane suggests a robust trend favoring DT as a cost-saving intervention, and the cost-effectiveness acceptability curve indicates a high probability that DT is cost-effective under typical willingness-to-pay thresholds. However, the economic evaluation was limited to a 12-week postoperative horizon and was based on very small differences in quality-of-life indices. Although DT appears cost-effective in the short term, a longer-term evaluation capturing downstream costs and quality-of-life changes would provide a more comprehensive assessment of its cost utility. Nevertheless, within the trial period, the probabilistic analysis suggests only a modest likelihood (20%-25%) that CT would be the more cost-effective option, reinforcing the conclusion that the DT system is an economically attractive alternative to standard care.

### Comparison With Prior Work

Both the DT and CT groups in the RCT achieved substantial gains in elbow flexion-extension ROM. This finding aligns with previous studies showing that DT can restore joint mobility as effectively as CT. For example, a pilot trial involving patients with elbow fractures found significant ROM improvements in both digital rehabilitation and clinic-based therapy, with no between-group difference [6]. Similarly, an integrative review of digital rehabilitation after orthopedic surgeries (primarily knee and hip arthroplasties) concluded that internet-based training yields improvements in joint ROM and other outcomes comparable to those achieved with standard in-person rehabilitation [16]. In other words, DT systems do not appear to impede recovery of motion, and existing evidence suggests that they achieve ROM gains equivalent to those of traditional therapy, consistent with the findings of our elbow stiffness trial.

Baseline forearm rotation differed between groups (*P*=.01), with the DT group demonstrating higher baseline values. Although the mixed-effects model included baseline forearm rotation as a covariate, which is the recommended approach for addressing regression to the mean, residual confounding due to this baseline imbalance cannot be entirely excluded. The adjusted between-group difference of approximately 13° (Table 3), therefore, reflects the estimated treatment effect after baseline correction. Nevertheless, this finding should be interpreted as exploratory because the trial was powered only for the noninferiority comparison of the primary end point. No formal MCID has been established for forearm rotation ROM in the published literature. Normal forearm rotation encompasses a total arc of approximately 160°, meaning that a 13° difference represents roughly 8% of normal motion. Whether this magnitude translates into a clinically meaningful functional benefit for patients remains uncertain. In addition, as discussed above, baseline forearm rotation differed between groups, and some portion of the observed difference may reflect residual imbalance despite statistical adjustment. Confirmatory studies specifically powered for this end point are needed to establish the clinical significance of this finding.

Recovery of muscle strength in the DT group was comparable to that in the CT group. This finding is supported by previous research in orthopedic rehabilitation. For example, following knee surgery, patients undergoing digital rehabilitation achieved strength gains indistinguishable from those of patients receiving clinic-based therapy [16]. Systematic reviews across various musculoskeletal conditions have likewise reported no significant differences in strength outcomes between digitally supervised exercise and CT when exercise protocols are comparable [16].

For patient-reported outcome measures, improvements in the DT group were comparable to those in the CT group. Previous mobile-based rehabilitation studies have consistently demonstrated equivalent functional outcome scores [6]. In a pilot study of elbow rehabilitation, both groups achieved significant functional gains, and the mobile-based rehabilitation group even showed a trend toward slightly better DASH scores early in recovery, although the differences were not statistically significant [6]. More broadly, a 2017 meta-analysis confirmed that mobile-based rehabilitation is as effective as usual care for improving physical function and reducing disability in musculoskeletal disorders [5]. In our trial, both groups also demonstrated improvements in quality of life, and prior evidence suggests that DT performs comparably to traditional training in this domain. Systematic reviews have reported that improvements in health-related quality of life are similar between DT and in-person care among patients with musculoskeletal conditions [17].

### Cost-Effectiveness

Our short-term cost-consequence analysis found that DT was associated with lower overall costs compared with conventional outpatient clinic-based training. The cost savings were driven primarily by reductions in primary care visits, secondary care costs, medication expenditures, and transportation expenses. These findings are strongly supported by previous cost-effectiveness studies [18-20]. A recent meta-analysis of 5 economic evaluations estimated that mobile-based rehabilitation was, on average, approximately US $90 less expensive per patient than standard rehabilitation for musculoskeletal conditions [18]. Another analysis of a mobile-based rehabilitation system after total knee arthroplasty reported cost savings of more than US $2400 per patient compared with traditional therapy, alongside equivalent or superior outcomes [19]. Our trial’s findings of lower costs favoring mobile-based rehabilitation are therefore consistent with prior evidence. Overall, DT systems appear to be cost-effective, providing similar health outcomes at lower or comparable costs relative to conventional in-person training.

### Adherence, Satisfaction, and Safety

Patient adherence to the DT system in the DT arm of the trial was high, reflecting a common trend reported in DT studies. Research indicates that patients often attend DT sessions and perform prescribed exercises as reliably as, or more reliably than, those receiving conventional therapy. A 2024 systematic review of RCTs found that patients undergoing mobile-based rehabilitation demonstrated approximately 9% higher exercise adherence on average compared with those receiving in-person care (95% CI 2%-16% higher) [21]. Attendance at scheduled sessions was also slightly better with DT systems, by as much as 8%, although the difference was not always statistically significant [21]. Likely contributing factors include convenience and flexibility, as patients can complete sessions from home, making adherence to the rehabilitation regimen easier. Patient satisfaction with the DT system in this trial was also very positive and may even have exceeded satisfaction with in-hospital therapy. This finding is consistent with previous studies reporting high acceptability of DT systems. In the elbow fracture pilot study, participants in the tele-rehabilitation group reported higher satisfaction with their care despite similar clinical outcomes [6]. Patients often value the comfort and individualized attention made possible through video sessions, and the therapeutic alliance can be maintained remotely. Importantly, studies have shown that when DT systems are delivered synchronously through real-time video, the quality of patient-therapist interaction is preserved, resulting in satisfaction ratings comparable to those of in-person visits [17]. Overall, the high adherence and satisfaction observed in the DT group are well supported by previous research, indicating that mobile-based rehabilitation is generally well accepted and achieves patient engagement comparable to that of conventional rehabilitation.

Regarding safety, the DT system used in this trial proved to be safe, with no increase in adverse events compared with CT. This finding is strongly consistent with existing evidence indicating that DT systems do not pose an increased risk to patients. A recent scoping review encompassing more than 80 mobile-based training studies across diverse populations found that the incidence of adverse events during DT sessions was extremely low, approximately 0.3% across 84,534 total sessions [22]. Importantly, most reported incidents were determined to be unrelated to the DT intervention itself [22].

### Clinical Implications

Within the context of this single-center trial, DT may be considered for integration into postsurgical rehabilitation pathways. Our study demonstrates that a DT system in which patients receive individually tailored PEST yields improvements in elbow ROM, strength, function, and quality of life comparable to those achieved with traditional outpatient clinic-based training, while also reducing overall costs. These findings support its use as a viable alternative, particularly for patients with limited access to specialized care or transportation challenges. The lower cost burden observed in our study, driven by reductions in primary care, secondary care, medication, and transportation expenses, further underscores its potential to reduce financial pressures on both patients and health care systems. In addition, the high levels of patient adherence and satisfaction, together with comparable safety profiles between the DT system and outpatient clinic-based training, suggest that digital interventions do not compromise treatment quality or clinical outcomes. Given these findings, clinicians may consider incorporating DT systems into standard postoperative rehabilitation protocols to enhance accessibility and optimize resource utilization. If validated in multicenter settings, DT systems may have broader applicability in musculoskeletal rehabilitation, although the generalizability of our single-center findings to other populations and health care systems requires further investigation [23-25]. Future efforts should focus on addressing barriers such as technology access and reimbursement policies to fully realize the benefits of DT systems in routine clinical practice.

### Strengths and Limitations

This study’s strengths include a rigorous randomized controlled design with an adequate sample size and comprehensive outcome measures encompassing clinical, functional, and economic end points. The noninferiority design allowed us to demonstrate that the DT system, in which patients receive individually tailored PEST, is as effective as conventional outpatient clinic-based training, while the inclusion of cost-effectiveness, adherence, and safety assessments provides a holistic view of the intervention’s impact. Moreover, high patient adherence and satisfaction underscore the feasibility and acceptability of the DT system in a postsurgical setting. However, several limitations must be acknowledged. First, our trial was originally powered for the primary end point of elbow ROM noninferiority, with 73 patients per group expected to provide 80% power (1-sided α=.025) to detect a 10° margin. The final sample (106 and 102 patients per group) exceeded this target, yielding approximately 90% power for the noninferiority hypothesis. This ample sample size strengthens confidence in the primary finding that DT is not inferior to CT for ROM improvement. However, as is typical in RCTs, our study was not specifically powered to detect small between-group differences in secondary outcomes. We examined multiple secondary end points (functional scores, strength measures, etc) and applied a Bonferroni correction to control type I error. This stringent adjustment, while guarding against false positives, inevitably lowers statistical power for each secondary comparison. Consequently, the chance of type II error is increased for secondary outcomes under our analysis strategy. In practical terms, our negative secondary results should be interpreted with caution: the trial may not have been large enough to uncover subtle advantages of either intervention in secondary domains. For example, given our final sample, the detectable difference in DASH score with 80% power at an adjusted α≈.007 is several points; smaller improvements could have gone undetected. We opted for a conservative approach to multiple comparisons to prioritize confidence in any positive findings, but we acknowledge that this comes at the expense of sensitivity. We have now supplemented the original Bonferroni correction with the Holm step-down procedure, which is uniformly more powerful for positively correlated end points, as recommended by both the Food and Drug Administration and the European Medicines Agency guidance. Both methods yielded identical conclusions regarding the secondary outcomes. Future studies with larger samples or without such stringent correction might further explore secondary benefits of DT. In our data, no secondary outcome showed a trend large enough to approach the adjusted significance threshold, which provides some reassurance that we did not miss any clinically meaningful effects. Nonetheless, the possibility of type II error for modest effects, particularly in patient-reported outcomes, remains a limitation of this study.

Second, a notable limitation is that we relied on self-reported measures for exercise adherence and patient satisfaction. Such self-reports are susceptible to social desirability bias, whereby participants may portray themselves in a favorable light. Research using concealed accelerometers has shown that self-reported exercise diaries can overestimate adherence by approximately 20% compared with objectively measured activity. In our trial, patients might have overestimated their adherence to the exercise regimen or overstated their satisfaction. Although the Joymotion app recorded exercise completion rates and session frequency, thereby providing a partial digital log, we did not use dedicated motion sensors or video verification to confirm exercise quality or dosage. The very high adherence scores should therefore be interpreted with caution. Future studies should incorporate wearable sensors or automated movement-quality assessment to complement patient reports. Consequently, although both groups reported excellent adherence (averaging 8.5/10) and satisfaction (9.5/10), these values should be interpreted with caution. It is possible that actual adherence was lower than reported, given evidence that individuals often overreport healthy behaviors. This limitation underscores the need for future studies to include objective monitoring of exercise completion to complement patient reports. Despite this caveat, the absence of between-group differences in self-reported adherence suggests that any social desirability bias was likely nondifferential between DT and CT and, thus, probably does not compromise the comparison of the 2 modalities, although absolute adherence levels may have been overestimated.

Third, the single-center design may limit the generalizability of our findings to other health care settings. Education level differed significantly between groups, and because education may correlate with digital literacy and comfort with technology, this imbalance could have influenced adherence and outcomes in the DT arm. A sensitivity analysis adjusting for education confirmed that the noninferiority conclusion was unchanged, but we cannot fully exclude residual confounding. The inability to blind participants and therapists introduces potential performance and expectation bias. This is particularly relevant for patient-reported outcomes such as ASES, DASH, and EQ-5D-5L scores, for which awareness of group assignment may have influenced response patterns. The small between-group differences observed in these measures may partly reflect equivalent patient expectations rather than purely equivalent treatment effects. Future trials should consider strategies to mitigate expectation bias, such as enhanced blinding of outcome assessors and the use of sham digital interfaces.

### Conclusions

This study showed that individually tailored PEST delivered via a DT system was statistically noninferior to conventional outpatient clinic-based training following arthroscopic release for posttraumatic elbow stiffness. Although noninferiority does not necessarily imply clinical interchangeability, the digital approach may represent a viable option for patients facing barriers to traditional in-person rehabilitation, provided that clinicians consider individual patient factors when selecting a rehabilitation modality.

## Appendix

Multimedia Appendix 1 CONSORT-eHEALTH (V 1.6.1).

Multimedia Appendix 2 The standardized 12-week physiotherapeutic elbow-specific training (PEST) protocol.

Multimedia Appendix 3 Unadjusted changes in outcomes for the digital training (DT) and conventional training (CT) groups at weeks 4, 12 and 24 after the surgery (per-protocol population).

Multimedia Appendix 4 Adjusted effectiveness estimates from linear mixed effects models (per-protocol population).

Multimedia Appendix 5 Sensitivity analysis of secondary outcomes at 12 weeks using Bonferroni and Holm step-down adjustments for multiple comparisons (intention-to-treat population).

## Abbreviations

ASES: American Shoulder and Elbow Surgeons
BTE: Baltimore Therapeutic Equipment
CONSORT: Consolidated Standards of Reporting Trials
CONSORT-NI: Consolidated Standards of Reporting Trials extension for noninferiority and equivalence trials
CT: conventional training
DASH: Disabilities of the Arm, Shoulder, and Hand
DT: digital training
ICER: incremental cost-effectiveness ratio
MCID: minimal clinically important difference
PEST: physiotherapeutic elbow-specific training
PT: physiotherapist
ROM: range of motion

## Data Availability

The deidentified data sets generated and analyzed during this study are available from the corresponding author on reasonable request. Requests should be directed to the corresponding author (SL) following online publication. All participants’ baseline characteristics and follow-up data are available in deidentified format.
