# Supplementary material for: Digital Physiotherapeutic Elbow-Specific Training System for Patients After Arthroscopic Release of Elbow Contracture: Noninferiority Randomized Controlled Trial
Source: JMIR Mhealth Uhealth. 2026 Jun 9;14:e87459. doi: 10.2196/87459 (PMC13291729; doi:10.2196/87459)
Supplement: Multimedia Appendix 2 [file mhealth_v14i1e87459_app2.docx]

**Multimedia Appendix 2.**

**Materials and Methods**

*Intervention*

Physiotherapeutic Elbow-Specific Training (PEST)

| **Week (Post-op)** | **Status & Key Assessments** | **Rehabilitation Content** | **Home Exercises** | **Adherence & Adjustments** |
| --- | --- | --- | --- | --- |
| **Week 1  (Immediate Post-op)** | **Pain & Swelling:** High immediately post-op; monitor pain (VAS) and edema.  **Wound:** Surgical dressings intact, no infection signs; suture line healing (stitches in place).  **ROM:** Severely limited pre-op; post-release arc improved but still restricted by pain.  **Neurovascular:** Check ulnar nerve sensation (due to traction during arthroscopy). | **Goal:** Prevent re-stiffening and protect healing tissues.  **Early mobilization:** Begin gentle, pain-free elbow ROM within 24–48 hours post-surgery​.  Emphasize flexion in first 1–2 weeks if surgeon recommends​, while moving into extension only to tolerance.  **Therapy sessions:** Daily or every other day PT in this first week if possible. Use active-assisted and passive elbow flexion/extension exercises (gravity-assisted flexion, gentle extension stretch).  **Adjacent joints:** Active wrist, hand, and shoulder ROM to maintain mobility and reduce swelling​.  **Pain management:** Cryotherapy (ice packs) after exercises and compression to control swelling​. Physician may prescribe NSAIDs (e.g. indomethacin 75 mg daily for 6 weeks to prevent heterotopic ossification​) and analgesics.  **Manual therapy:** Very gentle joint mobilizations (Grade I-II) for pain relief; soft tissue massage to reduce edema. | **Frequent ROM drills:** Hourly short-duration flexion-extension exercises (e.g. 5–10 minutes each hour while awake) within pain limits.  **Self-assisted stretches:** Use the opposite hand to assist bending and straightening the elbow to the point of stretch (5–6 sessions/day, 10–15 reps).  **Hand/forearm exercises:** Stress-ball squeezes and wrist bending/rotation exercises 3–5×/day to maintain strength.  **Shoulder mobility**: Pendulum swings or gentle shoulder elevation exercises daily to prevent shoulder stiffness. | **Considerations:**  **Protection:** A light removable splint or sling may be used for comfort between exercise sessions but remove it several times daily for motion exercises​. Avoid prolonged immobilization.  **Pain control:** Ensure pain is managed (meds 30 minutes before PT) so the patient can participate in exercise​.  **Frequency:** Emphasize near-hourly home ROM; patient/caregiver education is critical to prevent recurrent stiffness.  **Monitoring:** Educate patients to watch for warning signs (excessive pain, swelling, tingling) and to do only **non-painful ROM** to avoid inflammation. |
| **Week 2** | **Pain:** Improving but still present at end ranges; continue to track VAS pain with movement.  **Wound:** Skin healing well; suture removal around day 10–14 if not absorbable​.  **Swelling:** Moderating; mild effusion may persist.  **ROM:** Gradual gains – aim to approach functional arc (e.g. ~30–130°) or better. Goal is full passive flexion by end of week 2 if tolerated​. Extension likely still limited; measure degrees for progress. | **Goal:** Regain as much ROM as possible while initiating muscle activation.  **Stretching & mobilization:** Continue passive and active-assisted ROM in all planes (flexion, extension, pronation, supination) 3–5× daily. Add gentle prolonged stretching at end-range: e.g. low-load long-duration extension stretches using a towel or gravity (several minutes per session)​.  **Joint mobilizations:** Grade III oscillations or distraction techniques for extension if needed (posterior glides for flexion, anterior glides for extension) – within tolerance​.  **Scar management:** Once incision healed (~2 weeks), begin scar massage and elbow soft tissue mobilization to prevent adhesions.  **Muscle activation:** Initiate gentle isometric strengthening (pain-free): e.g. isometric biceps/triceps contractions, and light resistance for wrist flexors/extensors​.  **Modalities:** Continue icing after therapy and consider heat before sessions to warm tissues (if swelling is minimal). | **Home ROM program:** Continue aggressive home ROM exercises (active-assisted) at least 4–5 times/day​. Use gravity-assisted elbow extension (lying supine with arm overhead) and gentle self-overpressure into extension (~2 min hold, 3–4× daily)​.  **Self-stretch**: Extension stretch using other hand or resting arm on a pillow with a gentle weight for a few minutes. Flexion stretches by using the opposite hand to push forearm toward arm.  **Isometrics:** At home, perform pain-free isometric contractions (hold 5 seconds, 10 reps) for biceps, triceps, and forearm muscles, 2–3× per day.  **HEP log:** Patient keeps a log of daily exercise frequency to ensure compliance. | **Considerations:**  **No ROM restrictions:** Post-release, motion is allowed as tolerated (no structural repair to protect)​. Encourage reaching end-ranges but avoid aggressive force that causes sharp pain.  **Adherence:** The second week is critical – stress the importance of daily stretching (the major ROM gains are typically achieved in the first 4–6 weeks​).  **Customization:** If extension lags significantly behind flexion, consider a static progressive extension splint at night or dynamic brace to assist (optional, if standard exercises aren’t yielding gains)​.  **Therapy frequency:** 2–3 PT sessions/week to supervise stretching intensity, adjust techniques, and maintain motivation​. |
| **Week 3** | **Pain:** Generally mild at rest, moderate at end-range stretch; may still need occasional pain meds before therapy.  **ROM:** Improving; patient may have achieved functional flexion (~130°) and working on terminal extension (perhaps ~15–20° short of full). Pronation/supination nearly full if they were limited.  **Strength:** Slight atrophy of biceps/triceps from pre-op disuse; beginning to activate muscles. Grip strength improving if exercised.  **Functional:** Patient can do light ADLs (feeding, dressing) with less difficulty as ROM improves, but heavy tasks still limited. | **Goal:** Transition to active movements and begin light strengthening while continuing to improve ROM.  **Active ROM:** Progress to active-assisted ➔ active ROM in elbow flexion/extension as pain allows. Use active gravity-eliminated exercises (e.g. sliding arm on a table) progressing to against-gravity motions.  **Strengthening:** Initiate light isotonic strengthening for the elbow this week​. For example, begin biceps curls and triceps extensions with light dumbbells or resistance bands (e.g. 0.5–1 kg) for high-rep, low-load sets. Include forearm pronation/supination strengthening with light resistance (e.g. hammer rotations).  **Continued stretching:** After warm-up, continue focused end-range stretching – hold each stretch 30+ seconds. If lacking extension, therapist may apply prolonged passive stretch or use contract-relax techniques to gain last degrees.  **Shoulder & scapula:** Add scapular stabilizer exercises (like scapular pinches, serratus punches) and shoulder strengthening (rotator cuff isometrics) to support the overall arm function​.  **Neuromuscular control:** Begin **proprioceptive exercises** (e.g. elbow joint positioning drills, gentle closed-chain weight shifts) to re-integrate the elbow in functional patterns. | **Strength HEP:** Light dumbbell or Theraband exercises for elbow flexion/extension at home 3×/week (e.g. 2 sets of 15 reps at low weight). Maintain good form without pain.  **Stretching:** Continue daily ROM stretching routine – now emphasizing any remaining tight motion (often extension). For example, use a doorframe or wall to gently push the elbow into more extension stretch 2–3× daily (30–60s holds).  **Functional use:** Patient encouraged to use the arm in ADLs – e.g. grooming, eating, typing – to promote active functional ROM (within pain limits).  **Aerobic exercise:** Introduce lower-impact cardio that involves arms without heavy load (e.g. stationary bike or walking, gently swinging arms) to improve circulation and endurance. | **Considerations:**  **Weaning support:** By week 3, the sling is usually discontinued as comfort allows​, so the patient should try to keep the arm out of the sling to use it naturally.  **Pain management:** If stretching is very painful, short-term use of heat before exercise or analgesics can help. Emphasize that some discomfort is expected during stretching, but severe pain must be avoided​.  **Progression:** If ROM gains are slowing, therapists may increase intensity of mobilizations or add techniques like contract-relax. Conversely, if ROM is nearly full, shift more focus to strengthening while maintaining flexibility.  **Monitoring:** Continue weekly ROM measurements. By end of Week 3 or 4, aim for nearly full flexion and <10° extension lag if possible. Individual progress will vary; rehab plan may be adjusted accordingly. |
| **Week 4** | **Pain:** Minimal at rest; aches after therapy. Pain mainly at end-range extension if any.  **ROM:** Near full flexion likely (within a few degrees of opposite side). Extension still improving (perhaps ~10° short of full). Forearm rotation should be full or nearly full.  **Strength:** Elbow and forearm strength still ~30–50% weaker than the uninvolved side due to prior disuse but improving with exercise.  **Function:** Patient performing most light ADLs independently. Some difficulty with tasks requiring heavy lifting or end-range elbow extension (e.g. pushing up from a chair). | **Goal:** Achieve full elbow ROM and increase muscle strength/endurance.  **ROM & flexibility:** Continue aggressive end-range stretching to achieve full extension. May incorporate prolonged stretches (e.g. using a dynamic extension splint for 30 minutes if available, or therapist-applied 5-minute terminal stretch). Joint mob**s** as needed to address any remaining capsular tightness.  **Strength training:** Progress strengthening intensity: increase resistance slightly (e.g. move from 1 kg to 2 kg dumbbells as tolerated) for elbow flex/ext and forearm exercises. Begin resisted forearm supination/pronation (e.g. with light hammer or Theraband). Add shoulder strengthening (light dumbbell shoulder presses, rows) to integrate elbow use with shoulder.  **Endurance exercises:** Introduce light endurance training for the arm – e.g. UBE (arm cycle) with low resistance for 5–10 minutes​ to build tolerance for sustained activities.  **Functional movement:** Start gentle closed-chain exercises if appropriate: wall push-ups or table push-ups (partial weight) to encourage elbow extension under load (ensure no instability or pain).  **Manual therapy:** If scar tissue or myofascial tightness is palpable, continue soft tissue mobilization and begin more vigorous scar massage to mature the scar. | **Strength & endurance:** Home program now includes strength exercises 3–4 days/week (with a rest day between to allow recovery). Encourage higher repetitions (15–20 reps) to build endurance before focusing on pure strength.  **Stretch maintenance:** Continue daily stretching – even if full ROM is reached, keep stretching end-ranges to maintain it. For example, do a prolonged evening stretch into extension (using a weight on the wrist or lying face-up with arm overhead).  **Functional practice:** Patient to practice functional tasks at home that require elbow motion: e.g. reaching to a high shelf (elbow extension), lifting light objects (e.g. 1-2 kg) from floor to table (elbow flexion).  **Heat/ice as needed:** Use a heating pad before stretching at home if tight, and ice after exercise if swelling or soreness occurs. | **Considerations:**  **Milestone:** By ~4 weeks, many patients achieve full or nearly full passive ROM​. If patient has full ROM, shift goal to maintaining it and focusing on strengthening. If not, intensify stretching now before scar maturation makes gains harder.  **Adaptation:** Increase therapy exercises difficulty gradually (e.g. add slight resistance or new angles) to avoid plateau. But ensure no joint instability or pain with advanced exercises; if present, regress and strengthen more gradually.  **Patient education:** Reiterate that improvement slows over time – highest gains occur in early weeks​. Encourage patience and persistence, as even small weekly gains are meaningful.  **Clinical check:** Follow up with surgeon around 4-6 weeks to assess progress; the surgeon will expect substantial motion gains by now. Ensure communication about any concerns (e.g. abnormal pain or if extension still >30° limited). |
| **Week 5–6** | **Pain:** Generally low pain levels; may have mild soreness after exercises, but no significant pain during daily use.  **ROM:** Full flexion achieved; working on last few degrees of extension – patient may still lack ~5–10° (almost full straightening).  **Strength:** Improving; able to lift moderate light objects. By end of Week 6, expect ~70% strength return in elbow flexors/extensors, depending on pre-surgery muscle condition.  **Function:** Most ADLs pain-free. Can lift lighter groceries, perform personal care. Still regaining confidence in heavy or fast movements. | **Goal:** Solidify full ROM and advance to moderate strengthening and functional movements.  **Strength progression:** Moderate resistance strengthening now. Increase weight or resistance band level for biceps/triceps and wrist work (aim for fatigue at ~10–12 reps). Include eccentric exercises (e.g. slow lowering of weights) to build control. Forearm and grip strengthening with putty or grippers can be intensified.  **Dynamic exercises:** Begin more dynamic and multiplanar movements: e.g. light medicine ball tosses (two-handed initially) to engage elbow in coordination with shoulder. Incorporate PNF patterns diagonally involving the elbow (e.g. D2 flexion/extension patterns) to improve neuromuscular control​.  **Functional training:** **Simulate functional tasks** in clinic – e.g. reaching and lifting tasks, gentle pushing/pulling (light theraband rows, light bench press motions). If patient’s goals involve sports or manual work, tailor exercises (for example, mimic hammering motion, or throwing motion without weight).  **End-range focus:** If a slight extension deficit remains, continue end-range joint mobilizations and consider static progressive splinting at night to close the gap​.  **Endurance:** Increase cardiovascular exercise involving the arms: e.g. elliptical or light swimming movements (if wounds fully healed) to build endurance of the upper limb. | **Strength HEP:** Continue strengthening at home or gym. By week 6, patient can perform home strengthening 4–5×/week with gradually heavier resistance as tolerated (e.g. 2–3 kg weights for elbow exercises if able). They should incorporate both flexion/extension and forearm rotations.  **Stretching:** Nightly long-duration stretch for extension if any deficit remains (e.g. wear a static splint or use a weight on extended arm for 10 minutes). If full extension is achieved, still perform a brief stretch daily to maintain it.  **Functional use:** Encourage more use of the arm in household chores (light cleaning, carrying small loads) and work tasks (if applicable, with therapist guidance on safe techniques). Gradually reintroduce activities like driving (typically allowed ~6 weeks if pain-free and adequate control).  **PT homework:** If in a work-conditioning or work-hardening stage, follow specific drills provided by therapist to do on non-PT days. | **Considerations:**  **Phase transition:** By 6 weeks, the “acute” rehabilitation phase is ending​. The elbow should have minimal pain with motion and nearly full ROM, allowing a transition to more vigorous strengthening.  **Criteria to progress:** If patient has <10° extension loss and no swelling, they can safely advance strengthening​. If not, continue focused stretching before heavy strengthening.  **Patient confidence:** As strength returns, patient may start using the arm more normally. Emphasize proper body mechanics and avoiding compensatory movements (e.g. shoulder hiking) during tasks.  **Clinical:** If by 6 weeks significant stiffness persists (e.g. >30° motion loss), re-evaluate; more aggressive interventions (like dynamic splinting or a steroid injection) might be considered by the physician​. In most cases, however, continued therapy is pursued up to 12 weeks before such measures​ |
| **Week 7–8** | **Pain:** Minimal; typically only occasional soreness after heavy exercise. No rest pain.  **ROM:** Full elbow extension and flexion should be achieved or very close by this stage (0–140° or patient’s max). Pronation/supination full.  **Strength:** Near-normal for daily activities. Perhaps ~80% of contralateral side’s strength; remaining deficit in high-force or endurance tasks.  **Function:** Patient can perform most daily tasks normally. Possibly cleared for light work duties. Working on more demanding activities (sports, heavy labor tasks) as needed. | **Goal:** Restore full strength and begin higher-level functional training.  **Advanced strengthening:** Progress to heavier resistance and plyometric exercises as appropriate. For example, introduce push-ups (modified to full weight-bearing as tolerated), weighted ball throws or dribbling, and rapid elbow flex/extension drills to develop power. Ensure proper form and no elbow instability.  **PNF & agility:** Continue PNF patterns with increased resistance and speed. Add agility drills for the arm (for example, ladder drills for the hand or catching/light tossing to challenge elbow reaction and coordination).  **Functional simulation:** Incorporate work- or sport-specific exercises. If the patient is an athlete (e.g. racquet sports, baseball), begin sport-specific motion drills (shadow swinging a racket, mock throwing drills at submaximal intensity). If a manual worker, simulate lifting/carrying, using tools, etc., under supervision.  **Endurance:** Increase endurance challenges – e.g. longer duration arm bike or swimming laps, to ensure the elbow can tolerate sustained activity.  **Proprioception:** Add advanced proprioceptive training such as weight-bearing on an unstable surface (e.g. hands on a BOSU or wobble board) to enhance joint stability. | **Strength & power HEP:** Patient continues strengthening but can now include compound movements at home or gym (e.g. light dumbbell bench presses, overhead presses, kettle bell exercises) that involve the elbow. Perform these 2–3× per week, mixed in with prior elbow-specific exercises.  **Stretching:** At this stage, formal stretching can be reduced to a maintenance routine (e.g. brief stretches before and after workouts) if full ROM is already attained. If any tightness recurs, resume daily stretching as needed.  **Skill practice:** Encourage practice of specific skills relevant to patient’s goals outside of therapy. For example, gentle basketball free-throw practice, golf putting, or other low-stress practice that engages the elbow joint.  **Aerobic fitness:** If patient enjoys activities like swimming or biking, they can ramp up duration/intensity as cleared (these also help elbow endurance). | **Considerations:**  **Return to work/sport:** Clearance for work or sports is individualized. Many patients can start light sports or job tasks by ~8-12 weeks post-op, with full return by 3–6 months​. Ensure the patient demonstrates adequate strength and ROM required for their specific activities before full return.  **Joint protection:** Instruct on any necessary bracing or supports if returning to high-risk activities (though generally not needed if strength is recovered and ROM is full).  **Adaptive timing:** Some patients may reach this advanced phase sooner (week 7) while others may still be catching up on ROM. Adjust the program to the patient’s status – do not rush strengthening if ROM is still incomplete.  **Clinic visits:** If progress is good, therapy frequency might taper to 1x/week by week 8, focusing on checking form and progressing exercises, with patient largely independent in their workouts. |
| **Week 9–12** | **Pain:** Typically none or negligible. Patient may experience only occasional muscle soreness after strenuous activity.  **ROM:** Full ROM maintained (compare to opposite side; should be nearly symmetric in flexion/extension and forearm rotation).  **Strength:** Gradual return to baseline strength. By week 12, strength is often 90-100% of the uninvolved side for elbow motions (subjective and measured via manual testing or dynamometer).  **Function:** Patient should be functionally independent. Able to perform all usual ADLs and most work/sport tasks. Any remaining limitations are minor (perhaps slight stiffness after heavy use). | **Goal:** Achieve final strength gains and ensure a safe return to full function.  **Maximal strengthening:** In final weeks, push toward normal strength. Use higher resistances in exercises (as tolerated) aiming for last increments of strength. Include eccentric training for maximal strength (e.g. slow lowering of heavier weight than can lift up, under supervision).  **Plyometrics & power:** If appropriate to patient’s goals, include high-level plyometrics (clap push-ups, medicine ball rebounds) and power drills. These are typically for athletes or very active individuals and should be done only if elbow is pain-free and stable.  **Full functional retraining:** By now, patient transitions to activity-specific training. For athletes, begin controlled practice sessions of their sport with proper warm-up. For laborers, simulate full work demands in therapy (lifting heavy objects, using tools with force) to build confidence.  **Maintenance plan:** Develop a long-term exercise plan for the patient to continue post-discharge: a mix of stretching (to prevent recurrent tightness) and strengthening (to continue improving). Emphasize lifelong fitness of the arm to avoid future issues.  **Final assessment:** Perform outcome measures – e.g. goniometric ROM, strength testing, and functional scores (Mayo Elbow Performance Index, etc.) – to document improvements from baseline. Address any remaining deficits with targeted exercises in these last sessions. | **Independent gym program:** Patient should be transitioning to a self-managed routine. Encourage continuing strength workouts and stretching 2–3× weekly beyond therapy discharge to cement gains. Provide written instructions for any critical exercises.  **Stretching:** Advise the patient to integrate elbow stretches into their general fitness routine (especially if they notice morning stiffness or after heavy activity). E.g. a brief session of elbow ROM exercises daily or every other day.  **Home/work integration:** By 12 weeks, the patient is using the arm normally at home and work. They should continue to practice any challenging tasks at home to build more proficiency (for instance, practicing a musical instrument, gardening, or sport drills relevant to them).  **Self-monitoring:** Patient is taught to self-monitor for any signs of regression (increasing stiffness or pain) and to continue home exercises accordingly. | **Considerations:**  **Discharge planning:** Usually formal PT is concluded around 12 weeks post-op as patient meets goals​. Ensure patient has no pain with ADLs and full motion, and strength close to normal​.  **Follow-up:** Schedule a follow-up with the surgeon at 3 months to evaluate outcome; typically, by 3 months post-release, patients have achieved significant ROM gains (average ~30–40° arc improvement)​. If residual stiffness remains (e.g. >10° extension loss or functional limitation), the surgeon may consider interventions like a corticosteroid injection or manipulation under anesthesia​.  **Maintenance:** Instruct that improvements can continue up to 6–12 months post-op with persistent exercise​. The patient should maintain a home program to preserve gains. Even minor ROM improvements (a few degrees) beyond 3 months can enhance function and are worth pursuing​.  **Outcome:** By 12 weeks, most patients have markedly better elbow function and little to no pain​. They can resume full activities, though return to high-level sports or maximal strength might occur in subsequent months as they continue conditioning​ |

*Outcome Measures*

Isometric flexion strength, dynamic flexion strength: Isometric flexion strength and dynamic flexion strength of the operative side were measured and compared with the contralateral side using a BTE machine (Baltimore Therapeutic Equipment, Simulator II, Hanover, MD, USA). All measurements were performed bilaterally first on the non-surgical side and then on the surgical side under the following protocol:

- 1. a. The BTE machine was adjusted to correct lever arm length and isometric flexion strength was measured first. With the elbow flexed at 90° and the hand grasping the lever arm in supinated position, patients were asked to flex the lever arm as hard as possible and hold for six seconds. Patients were asked to perform this procedure two more times for a total of three trials. The strength measurement reported is the mean of 3 trials with a coefficient of variation < 10%.
  2. b. Dynamic flexion strength was assessed with 50% of the peak isometric flexion strength recorded in the previous step moving through full ROM as fast as possible for 10 seconds. No resistance was set in extension. Dynamic strength was recorded in Engals as a function of power (work/time) (1 Engal is the effort required to move a load of 1 inch-pound 1° in 1 sec).

**References**

1. Adolfsson L. Post-traumatic stiff elbow. EFORT Open Rev. 2018;3(5):210-216.
2. Attum B, Obremskey W. Posttraumatic Elbow Stiffness: A Critical Analysis Review. JBJS Rev. 2016;4(9):e1.
3. Park MJ, et al. Surgical Release for Posttraumatic Loss of Elbow Flexion. JBJS Essent Surg Tech. 2011.
