# Supplementary material for: Digital Physiotherapeutic Elbow-Specific Training System for Patients After Arthroscopic Release of Elbow Contracture: Noninferiority Randomized Controlled Trial
Source: JMIR Mhealth Uhealth. 2026 Jun 9;14:e87459. doi: 10.2196/87459 (PMC13291729; doi:10.2196/87459)
Supplement: Multimedia Appendix 3 [file mhealth_v14i1e87459_app3.docx]

**Multimedia Appendix 3. Unadjusted changes in outcomes for the DT and CT groups at weeks 4, 12 and 24 after the surgery (per-protocol population)**

| **Outcome*** | **4 weeks post-surgery** | | | **12 weeks post-surgery** | | | | **24 weeks post-surgery** | | | |
| --- | --- | --- | --- | --- | --- | --- | --- | --- | --- | --- | --- |
|  | **DT group (N=100)** | **CT group (N=96)** | **P value** | | **DT group (N=102)** | **CT group (N=96)** | **P value** | | **DT group (N=100)** | **CT group (N=95)** | **P value** |
| **ROM of elbow flexion to extension motion (°)** | 9.36 (12.58) | 10.17 (14.77) | 0.691 | | 19.47 (15.30) | 20.89 (17.38) | 0.556 | | 22.82 (15.13) | 24.22 (16.61) | 0.550 |
| **ROM of forearm rotation (°)** | 25.96 (20.49) | 26.39 (20.29) | 0.886 | | 43.24 (25.07) | 46.67 (22.02) | 0.327 | | 52.07 (26.64) | 56.44 (23.96) | 0.244 |
| **Flexion Strength - Isometric Elbow Flexion Strength (%)** | 5.27 (6.10) | 5.01 (6.10) | 0.767 | | 21.53 (6.04) | 20.41 (6.09) | 0.213 | | 25.93 (5.35) | 24.38 (5.40) | 0.053 |
| **Flexion Strength - Dynamic Elbow Flexion Strength (%)** | 5.11 (5.87) | 4.25 (4.79) | 0.280 | | 17.01 (5.58) | 16.53 (5.28) | 0.549 | | 23.29 (5.42) | 23.04 (5.38) | 0.753 |
| **ASES Function Subscore (points)** | 4.31 (2.89) | 3.98 (2.99) | 0.447 | | 8.72 (3.89) | 8.23 (3.95) | 0.392 | | 6.45 (4.29) | 5.93 (4.11) | 0.397 |
| **ASES Pain Subscore (points)** | -4.99 (4.41) | -5.67 (4.49) | 0.303 | | -12.72 (6.51) | -13.12 (6.70) | 0.684 | | -10.90 (6.55) | -11.26 (6.94) | 0.717 |
| **DASH Score (points)** | -11.97 (7.70) | -12.50 (7.09) | 0.627 | | -24.94 (9.36) | -25.49 (8.98) | 0.684 | | -26.18 (9.22) | -26.59 (8.77) | 0.758 |
| **EQ-5D-5L** | 0.00 (0.00) | 0.00 (0.00) | / | | 0.08 (0.05) | 0.08 (0.06) | 0.563 | | 0.08 (0.05) | 0.09 (0.06) | 0.563 |

DT: digital training system; CT: conventional outpatient clinic-based training; ROM: range of motion; ASES: American Shoulder and Elbow Surgeons Shoulder Score; DASH: The disabilities of the arm, shoulder and hand questionnaire. EQ-5D-5L: EuroQoL 5-Dimension 5-Level

Values represent the mean change from baseline for each group, reported as mean (standard deviation), with P values comparing between-group differences at each follow-up point. All outcome measures were unadjusted.

*Isometric flexion strength and dynamic flexion strength were measured and compared with the contralateral side using a BTE machine (Baltimore Therapeutic Equipment, Simulator II, Hanover, MD, USA)
