# Supplementary material for: Digital Physiotherapeutic Elbow-Specific Training System for Patients After Arthroscopic Release of Elbow Contracture: Noninferiority Randomized Controlled Trial
Source: JMIR Mhealth Uhealth. 2026 Jun 9;14:e87459. doi: 10.2196/87459 (PMC13291729; doi:10.2196/87459)
Supplement: Multimedia Appendix 4 [file mhealth_v14i1e87459_app4.docx]

**Multimedia Appendix 4. Adjusted effectiveness estimates from linear mixed effects models (per-protocol population)**

| **Outcome*** | **4 weeks post-surgery** | | | **12 weeks post-surgery** | | | **24 weeks post-surgery** | | |
| --- | --- | --- | --- | --- | --- | --- | --- | --- | --- |
|  | **Coefficient** | **95% CI** | **P value** | **Coefficient** | **95% CI** | **P value** | **Coefficient** | **95% CI** | **P value** |
| **ROM of elbow flexion to extension motion (°)** | -2.142 | (-3.694, -0.590) | 0.007 | -2.527 | (-3.699, -1.356) | 0.000 | -2.715 | (-3.621, -1.808) | 0.000 |
| **ROM of forearm rotation (°)** | 13.100 | (1.589, 24.612) | 0.026 | 12.135 | (9.982, 14.289) | 0.000 | 11.430 | (9.374, 13.486) | 0.000 |
| **Flexion Strength - Isometric Elbow Flexion Strength (%)** | -0.324 | (-1.009, 0.360) | 0.353 | -0.094 | (-0.598, 0.411) | 0.716 | 0.199 | (-0.476, 0.875) | 0.564 |
| **Flexion Strength - Dynamic Elbow Flexion Strength (%)** | -0.228 | (-0.995, 0.539) | 0.560 | -0.112 | (-0.644, 0.421) | 0.681 | -0.166 | (-0.557, 0.225) | 0.406 |
| **ASES Function Subscore (points)** | 0.785 | (-0.154, 1.723) | 0.101 | 0.928 | (0.594, 1.262) | 0.000 | 1.030 | (0.721 , 1.338) | 0.000 |
| **ASES Pain Subscore (points)** | 0.291 | (-0.666, 1.249) | 0.551 | 0.391 | (-0.044, 0.826) | 0.078 | 0.457 | (0.123, 0.791) | 0.007 |
| **DASH Score (points)** | -0.224 | (-1.959, 1.510) | 0.800 | -0.138 | (-0.540, 0.265) | 0.503 | -0.119 | (-0.406, 0.168) | 0.417 |
| **EQ-5D-5L** | -0.003 | (-0.015, 0.009) | 0.604 | -0.005 | (-0.009, -0.001) | 0.010 | -0.006 | (-0.010, -0.003) | 0.000 |

ROM: range of motion; ASES: American Shoulder and Elbow Surgeons Shoulder Score; DASH: The disabilities of the arm, shoulder and hand questionnaire. EQ-5D-5L: EuroQoL 5-Dimension 5-Level.

Each coefficient represents the estimated between-group difference in the change from baseline (Intervention group minus Control group) for the specified outcome at that follow-up time point. Positive coefficients indicate higher scores in the Intervention group compared to the Control group, whereas negative values indicate lower scores in the Intervention group. Each estimate is presented with its 95% confidence interval (CI) and corresponding P value. All outcome measures were adjusted for baseline values in the model.

*Isometric flexion strength and dynamic flexion strength were measured and compared with the contralateral side using a BTE machine (Baltimore Therapeutic Equipment, Simulator II, Hanover, MD, USA)
