# Supplementary material for: Digital Physiotherapeutic Elbow-Specific Training System for Patients After Arthroscopic Release of Elbow Contracture: Noninferiority Randomized Controlled Trial
Source: JMIR Mhealth Uhealth. 2026 Jun 9;14:e87459. doi: 10.2196/87459 (PMC13291729; doi:10.2196/87459)
Supplement: Multimedia Appendix 5 [file mhealth_v14i1e87459_app5.docx]

**Multimedia Appendix 5.** Sensitivity Analysis of Secondary Outcomes at 12 Weeks Using Bonferroni and Holm Step-Down Adjustments for Multiple Comparisons (Intention-to-Treat Population)

| **Outcome** | **Adjusted Difference (DT minus CT)** | **95% CI** | **Unadjusted P Value** | **Bonferroni Adjusted Pᵇ** | **Holm Adjusted Pᶜ** | **Significance (Holm)** |
| --- | --- | --- | --- | --- | --- | --- |
| ***Primary endpoint*** |  |  |  |  |  |  |
| Elbow flexion-extension ROM, deg | -2.81 | -5.31 to -0.31 | .03 | NA | NA | Noninferiorᵈ |
| ***Secondary endpoints*** |  |  |  |  |  |  |
| Forearm rotation ROM, deg | 13.317 | 10.422 to 16.213 | <.001 | <.001 | <.001 | Yes |
| Isometric flexion strength, % contralateral | -0.073 | -0.539 to 0.393 | .76 | >.99 | >.99 | No |
| Dynamic flexion strength, % contralateral | 0.136 | -0.487 to 0.759 | .67 | >.99 | >.99 | No |
| ASES function subscore, points | 0.644 | 0.327 to 0.960 | <.001 | <.001 | <.001 | Yes |
| ASES pain subscore, points | 0.298 | -0.133 to 0.729 | .18 | >.99 | .70 | No |
| DASH score, points | 0.025 | -0.270 to 0.320 | .87 | >.99 | >.99 | No |
| EQ-5D-5L utility index | -0.003 | -0.006 to -0.001 | .003 | .02 | .02 | Yes |

CI, confidence interval; CT, conventional training; DT, digital training; ROM, range of motion; ASES, American Shoulder and Elbow Surgeons; DASH, Disabilities of the Arm, Shoulder and Hand; EQ-5D-5L, EuroQol 5-Dimension 5-Level.

Values are presented as mean (SD) at 12 weeks postoperatively. Adjusted differences were estimated from linear regression models adjusting for baseline value of the respective outcome, age, and sex. Positive values favor the DT group (higher score) except for ASES pain and DASH where lower scores indicate better outcomes.

ᵃ Adjusted differences are from linear mixed-effects models with fixed effects for group, time, group-by-time interaction, age, sex, and baseline value of the respective outcome, with random intercepts for subjects. Coefficients represent the group-by-time interaction at 12 weeks (DT minus CT). Values are consistent with Table 4 of the manuscript.

ᵇ Bonferroni correction: each secondary endpoint tested at alpha = .05/7 = .0071. Three endpoints met this threshold: forearm rotation (P<.001), ASES function (P<.001), and EQ-5D-5L (P=.003).

ᶜ Holm step-down procedure: P values are ranked from smallest to largest and tested sequentially against alpha/(k-j+1), where k=7 and j is the rank. Testing stops at the first non-rejected hypothesis. The Holm method controls the familywise error rate identically to Bonferroni while being uniformly more powerful.

ᵈ Primary outcome was assessed using a separate noninferiority test (one-sided alpha=.025, pre-specified 10-degree margin) and was not subject to multiplicity adjustment. The upper bound of the 95% CI (--0.31 deg) did not exceed the margin, confirming noninferiority.

Holm Step-Down Sequential Testing Procedure

| **Step** | **Outcome (ordered by P)** | **Unadjusted P Value** | **Holm Multiplier (k minus j + 1)** | **Holm Adjusted P** | **Sequential Threshold (alpha / multiplier)** | **Decision** |
| --- | --- | --- | --- | --- | --- | --- |
| 1 | Forearm rotation, deg | 1.98e-19 | 7 | 1.39e-18 | 0.00714 | Reject H₀ |
| 2 | ASES function subscore, points | 6.66e-05 | 6 | 4.00e-04 | 0.00833 | Reject H₀ |
| 3 | EQ-5D-5L index | 0.0030 | 5 | 0.0150 | 0.01000 | Reject H₀ |
| 4 | ASES pain subscore, points | 0.1750 | 4 | 0.7000 | 0.01250 | Fail to reject |
| 5 | Dynamic flexion strength, % | 0.6690 | 3 | >.99 | 0.01667 | Fail to reject |
| 6 | Isometric flexion strength, % | 0.7590 | 2 | >.99 | 0.02500 | Fail to reject |
| 7 | DASH score, points | 0.8680 | 1 | 0.8680 | 0.05000 | Fail to reject |
